# Intestinal epithelial cell NCoR deficiency ameliorates obesity and metabolic syndrome

**DOI:** 10.1016/j.apsb.2024.09.019

**Published:** 2024-10-10

**Authors:** Shaocong Hou, Hengcai Yu, Caihong Liu, Andrew M.F. Johnson, Xingfeng Liu, Qian Jiang, Qijin Zhao, Lijuan Kong, Yanjun Wan, Xiaowei Xing, Yibing Chen, Jingwen Chen, Qing Wu, Peng Zhang, Changtao Jiang, Bing Cui, Pingping Li

**Affiliations:** a State Key Laboratory of Bioactive Substance and Function of Natural Medicines, Institute of Materia Medica, Chinese Academy of Medical Sciences and Peking Union Medical College, Beijing 100050, China; b Diabetes Research Center of Chinese Academy of Medical Sciences, Beijing 100730, China; c CAMS Key Laboratory of Molecular Mechanism and Target Discovery of Metabolic Disorder and Tumorigenesis, Beijing 100050, China; d Department of Physiology and Pathophysiology, School of Basic Medical Sciences, Peking University, Beijing 100191, China; e Division of Metabolic and Bariatric Surgery, Department of General Surgery, Beijing Friendship Hospital, Capital Medical University, National Clinical Research Center for Digestive Diseases, Beijing 100050, China; f The Access to Advanced Health Institute, Seattle, Washington 98102, USA

**Keywords:** Nuclear receptor co-repressor 1, Intestinal epithelium, Metabolic syndrome, PPAR alpha, Liver X receptor, Thermogenesis, Succinate, Cholesterol

## Abstract

Nuclear receptor corepressor (NCoR1) interacts with various nuclear receptors and regulates the anabolism and catabolism of lipids. An imbalance in lipid/energy homeostasis is also an important factor in obesity and metabolic syndrome development. In this study, we found that the deletion of NCoR1 in intestinal epithelial cells (IECs) mainly activated the nuclear receptor PPAR*α* and attenuated metabolic syndrome by stimulating thermogenesis. The increase in brown adipose tissue thermogenesis was mediated by gut-derived tricarboxylic acid cycle intermediate succinate, whose production was significantly enhanced by PPAR*α* activation in the fed state. Additionally, NCoR1 deletion derepressed intestinal LXR, increased cholesterol excretion, and impaired duodenal lipid absorption by decreasing bile acid hydrophobicity, thereby reversing the possible negative effects of intestinal PPAR*α* activation. Therefore, the simultaneous regulatory effect of intestinal NCoR1 on both lipid intake and energy expenditure strongly suggests that it is a promising target for developing metabolic syndrome treatment.

## Graphical abstract

Intestinal NCoR1 deficiency activated PPAR*α* and LXR signaling, which increased succinate production, enhanced cholesterol excretion, and decreased bile acid hydrophobicity, thereby stimulating energy expenditure and inhibiting lipid absorption.

**Figure:**
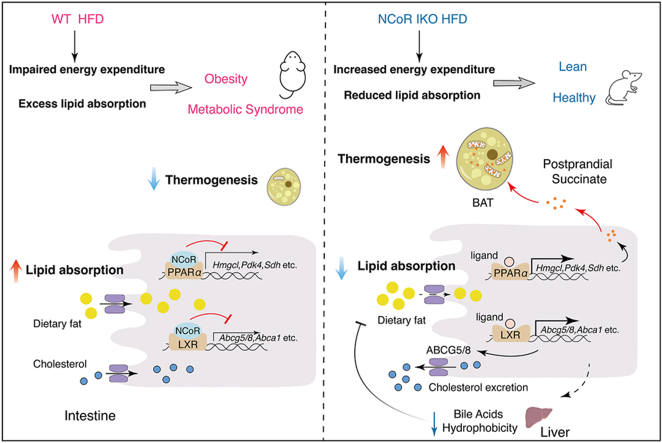

## 1 Introduction

Metabolic syndrome is a cluster of risk factors consisting of visceral obesity, hyperglycemia, dyslipidemia, and hypertension^1^. These factors also increase the incidence of many other pathologies, including cardiovascular disease, type 2 diabetes (T2D), and metabolic-associated fatty liver disease^2^. One major driver of metabolic syndrome is the imbalance of energy homeostasis, which is characterized by the intake of excess fat and relatively insufficient fat burning through physical exercise and adaptive thermogenesis^3^.

The anabolism and catabolism of lipids are mainly controlled by nuclear receptors (NRs), including peroxisome proliferator-activated receptors (PPARs) and liver X receptor (LXR). NRs represent a class of transcription factors regulated by ligands like lipid metabolites and control various biological processes in cell metabolism^4^. These effects are exerted through direct regulation of the respective target genes. For example, the PPAR*α* isoform of PPARs positively regulates multiple genetic pathways, including fatty acid (FA) uptake, FA oxidation, and ketogenesis. As such, it's an established target of the hypolipidemic drugs fibrates^5^. PPAR*α* agonist administration in rodents can also suppress diet-induced obesity and upregulate thermogenic genes in brown adipose tissue^6^. Therefore, the regulation of NRs and their target genes has meaningful impacts on the energy balance.

NRs regulate their target genes by recruiting coregulators, including corepressors and coactivators. Nuclear receptor corepressor (NCoR1) is a basal component of the corepressor complex and represses the activities of NRs in the absence of ligands^7^. The deletion of NCoR1 has been found to regulate certain types of NRs in different tissues. Both PPAR*γ* activation in adipose tissue^8^ and LXR activation in macrophages^9^ improve insulin resistance, whereas the hepatic mutation of NCoR1 decreases intestinal cholesterol absorption in a thyroid hormone receptor-dependent manner^10^. Thus, the interaction of NCoR1 with NRs is variable and dependent on the tissue type. The intestinal-specific deletion of NCoR1 in a hyperbilirubinemia model derepresses both the PPAR*α* and LXR signaling pathways, as shown by RNA-seq and ChIP-seq analyses^11^. However, whether intestinal NCoR1 can affect metabolic homeostasis similarly to NCoR1 in other tissues is unexplored.

Here, we demonstrated that the deletion of NCoR1 in intestinal epithelial cells (IECs) prevented mice from developing diet-induced metabolic syndrome and obesity. These effects were mainly mediated by intestinal PPAR*α* activation, which promoted the postprandial production of the tricarboxylic cycle intermediate succinate and stimulated thermogenesis. Moreover, the unwanted effects of PPAR*α* on lipid absorption were counteracted by intestinal LXR de-repression, which induced cholesterol excretion and decreased bile acid (BA) hydrophobicity, thereby reducing lipid absorption. Collectively, these results showed that NCoR1 can serve as an energy switch that regulates both energy intake and expenditure.

## 2 Materials and methods

### 2.1 Conditional intestinal epithelial cell NCoR1 knockout mice

NCoR1 *flox*/*flox* (WT) mice were generated as previously described^8^. For intestine epithelial cell (IEC)-specific knockout, mice harboring the villin promoter-controlled Cre recombinase were crossed with NCoR1 *flox*/*flox* (WT) mice to obtain conditional IEC NCoR1-knockout (IKO) mice. The animals were housed in a 12-h/12-h light-dark cycle animal facility. Food and water are accessible freely. Seven- to eight-week-old age- and sex-matched male WT and IKO mice were fed with chow or a high-fat diet (60% kcal from fat, D12492, Research Diets, Inc.,) for a designated time in each experiment. The body weight was recorded on a weekly basis. Fat and lean mass of non-anesthetized mice were measured with an echo-MRI body composition measurement system (QMR06-090H, NIUMAG, Suzhou, China). After HFD feeding, the mice were sacrificed, the intestines were separated, the intestines were washed with saline to remove luminal contents, the intestines were cut longitudinally, and the intestinal mucosa was carefully scraped. Liver, WAT, and pancreas tissue were separated and weighed. Muscle and BAT were also collected. After being snap-frozen in liquid nitrogen, all tissues were transferred to a −80 °C freezer. For the time-course LXR target gene expression experiments, age- and sex-matched C57BL/6J mice were divided into two groups and fed either a chow diet or an HFD for 32 weeks. At the designated time points, mice from each diet group were killed, and intestinal tissues were collected. We only used male mice. All procedures for animal experiments were approved by the Committee of Institute of Material Medica, Chinese Academy of Medical Sciences and Peking Union Medical College (No. 00008823).

### 2.2 Inducible conditional intestinal NCoR1-knockout mice

Villin-Cre^ERT2^ mice were obtained courtesy of Dr. Robine Sylvie from Curie Institute, Paris, and Dr. Rongwen Xi from NIBS, Beijing. Inducible IEC NCoR1-knockout (*NCoR1*^*ΔIECi*^) mice were obtained by crossing NCoR1 *flox*/*flox* (*NCoR1*^*f/f*^) mice with Villin-Cre^ERT2^ mice. Male mice were used. Seven- to eight-week-old age- and sex-matched mice were chosen and fed with HFD (D12492) to the end. NCoR1 deletion was initiated by injecting tamoxifen (100 mg per mouse, Sigma–Aldrich) after 6 weeks of HFD feeding. Tamoxifen was injected for 5 days, and the mice were used in subsequent studies after 2 weeks resting.

### 2.3 Insulin tolerance tests (ITTs), glucose tolerance tests (GTTs), and hyperinsulinemic euglycemic clamp studies

For GTTs, WT and IKO mice were fasted for 6 h before gavage of glucose (concentration: 2 g/kg). At 0, 15, 30, 60, 90 and 120 min after glucose administration, blood was collected for measurements of blood glucose levels with a glucometer (Accuchek, Roche).

For ITTs, insulin (0.3 units/kg, Humulin R, Eli Lily, and Company) was injected subcutaneously after 6 h of fasting. After insulin injection, blood glucose levels were then measured at 0, 15, 30, 60, 90 and 120 min with a glucometer (Accuchek, Roche).

Hyperinsulinemic-euglycemic clamps were carried out as previously described^12^. Briefly, the right jugular vein of the anesthetized mice was exposed, and one catheter (Silastic 508-001, Dow Corning) was implanted, tunneled to the back of the neck subcutaneously, and exteriorized. After 3 days of recovery, their body weights were measured, and mice that lost >10% of their pre-surgery weight were excluded from the clamp experiments. After a 6-h fast, blood was collected for blood glucose level determination and stored for measurements of plasma free fatty acid (FFA) and insulin. Then d-[3-^3^H] glucose (NET331C, PerkinElmer Life Sciences) was infused at a constant rate of 5 μCi/h for 90 min to achieve tracer equilibration. Blood samples were collected to measure basal glucose turnover. Subsequently, at *t* = 0 min, insulin (12 mU/kg/min, Humulin R) mixed with tracer (5 μCi/h) was infused at a constant rate into the jugular vein, and the infusion of 50% d-glucose was started at a low rate. Blood glucose levels were measured from tail vein blood at 10-min intervals, and the glucose infusion rate was increased gradually until the steady-state conditions (120 ± 5 mg/dL) were achieved. Steady state was confirmed by maintaining the glucose infusion rate and blood glucose levels within constant ranges for a minimum of 30 min. At *t* = 0 min (basal), *t* = 110 min, and *t* = 120 min (end of the experiment), blood samples were collected for glucose-specific activity determination (MicroBeta2 microplate counter, PerkinElmer Life Sciences) and measurement of the FFA and insulin concentrations. The Steele equation was used to calculate tracer-determined rates. The assumption is that the glucose disappearance rate (total GDR) is equivalent to the combined rate of endogenous glucose production (HGP) and the exogenous glucose infusion rate (GIR). Whereas the insulin-stimulated glucose disposal rate (IS-GDR) is calculated as the glucose disappearance rate minus the basal glucose turnover rate.

### 2.4 Insulin analysis

To measure plasma insulin levels, 6 h fasted mice were orally administered with 2 g/kg glucose and blood was sampled at the fasted state and 15 min post glucose administration. Plasma insulin levels were determined with ELISA (Alpco, 80-INSMSU-E10).

### 2.5 Lipid measurement

Mice were fasted for 6 h, and blood samples were drawn from tail vein to measure the triglyceride (TG), total cholesterol (TC) and FFA levels. The TG and TC levels were measured using kits from BioSino Bio-Technology and Science Incorporation, and the FFA levels were measured using Wako kits (290-63701, Wako LabAssay). The levels of high-density lipoprotein cholesterol (HDL-c) and low-density lipoprotein/very low density lipoprotein cholesterol (LDL/VLDL-c) were measured with assay kits from Cell Biolabs (STA-391, Cell Biolabs, Inc.).

For the assessment of tissue lipids, 20 mg tissue (liver, duodenum, jejunum, and ileum) was homogenized with 5% Triton X-100 saline at room temperature. After homogenization, the hepatic TG levels and the hepatic and intestinal TC levels were measured using kits from BioSino Bio-Technology and Science Incorporation. Serum Triglyceride Determination Kit (TR0100, Sigma–Aldrich) was used to determine the intestinal TG levels, and Wako kits (290-63701, Wako LabAssay) were used to measure FFA levels.

For the analysis of fecal lipids, feces collected from individually housed mice for 24 h were dried (60 °C), weighed and ground. Lipids were extracted from 50 mg of powder using methanol:chloroform (1:2, *v*/*v*) at 37 °C with vigorous shaking for 12 h. The pellets were dissolved in isopropanol supplemented with 10% Triton X-100. TG, TC and FFA levels were measured as above. The fecal lipid content was calculated as Eq. (1):

(1) The fecal lipid content = [((Concentration lipid/100 × 0.5)/1) × 4 × (24-h Total feces/Feces used)]/Body weight

For biliary cholesterol analysis, 3 μL of bile and 45 μL of Milli-Q water were mixed and added to 200 μL of chloroform:methanol (2:1) for lipid extraction. After separation, the organic phase was collected, air-dried and redissolved in isopropanol. Cholesterol levels were determined with a commercial kit (BioSino Bio-Technology and Science Incorporation).

### 2.6 Analysis of FAs in BAT

BAT was thawed on ice and weighed. Then the tissues were homogenized with 400 μL of methanol on ice, and 10 μL of homogenized sample were transferred to a new tube. After the addition of 40 μL of MeOH, the mixture was vortexed for 1 min, then centrifuged for 10 min at 14,000 rpm, 4 °C (5430R, Eppendorf, Hamburg, Germany). Thirty microliters of the supernatant were diluted with 30 μL of H_2_O (*v*/*v*, 50/50). Two microliters of pretreated sample solution were used, and the detection was performed on an ultra-performance liquid chromatograph coupled with a triple quadrupole mass spectrometry system (UPLC–MS/MS, I-Class-Xevo TQ-s Micro, Waters, USA).

Negative electrospray ionization (ESI-) mode was used in analysis, and separation of FFAs was on an HSS T3 column (2.1 mm × 30 mm, 1.8 μm). Two mobile phases were used: A was 0.01% formic acid in H_2_O and 0.2 mmol/L NH_4_HCO_2_, and B was 50% isopropanol in acetonitrile mixed with 0.01% formic acid and 0.2 mmol/L NH_4_HCO_2_. The FFAs were eluted and separated with a gradient of 50%–98% mobile phase B over 1.2 min. The flow rate was 1.3 mL/min. Then the column was washed with 98% mobile phase B for 0.5 min and re-equilibrated to the initial conditions.

### 2.7 Lipid absorption analysis

TG tolerance test was conducted on mice fasted overnight (19:30 pm–9:30 am) by oral gavage of olive oil (10 μL/g body weight). Blood was collected before olive oil gavage and 1, 2, 3 and 4 h after gavage. For the LPL-mediated inhibition of lipid absorption, overnight fasted mice were intraperitoneally injected with poloxamer 407 (1 g/kg, Sigma–Aldrich) and then administered with 200 μL of olive oil. Blood was collected before (0 h) and at 1, 2, 3, and 4 h after olive oil gavage. For the fasting-refeeding experiment, the mice were fasted overnight and refed with HFD for 12 h. At 0, 6 and 12 h after refeeding, blood was drawn from the tail. The plasma TG levels were measured with a triglyceride determination kit (TR0100, Sigma–Aldrich).

Oil Red O staining was performed on overnight-fasted mice. After gavage with 10 μL/g olive oil, all mice were sacrificed, and the duodenum was separated and washed. The tissue was embedded in OCT and sliced into frozen sections (10 μmol/L), then stained for Oil Red O with commercial kit (G1015; Servicebio).

### 2.8 Immunohistochemistry

The small intestine was segmented into three parts: duodenum, jejunum, and ileum. All three parts were washed with saline and fixed in 4% neutral paraformaldehyde. For H&E staining, the liver and epididymal adipose tissue were also fixed, embedded in paraffin and sectioned. The villus-crypt length was measured, and the cells were counted using ImageJ.

### 2.9 Indirect calorimetry and rectal temperature measurement

The HFD-fed WT and IKO mice were placed in an environment-controlled 12-chamber comprehensive lab animal monitoring system (CLAMS, Columbus Instruments, Columbus, OH, USA). The mice were allowed to acclimatize for 48 h and then monitored at 23 °C for 24 h. Food and water were freely accessed. An 8-chamber CLAMS was used for the succinate sodium-treated mice. The acclimatization period was 24 h, and then the mice were monitored at 23 °C for 72 h.

A digital thermometer with attached rectal probe (Physitemp) was used to measure rectal temperatures of the HFD-fed WT and IKO mice.

### 2.10 Intestinal permeability measurement

For the assessment of intestinal barrier function, an *in vivo* permeability assay was conducted. Food was withdrawn from the mice for 6 h, and then, FITC-dextran (Sigma, FD4, 45 mg/100 g BW) was given orally. After 4 h, blood was collected with heparin-coated microtubes and centrifuged at 1000 × *g* for 10 min. After dilution with PBS (1:10), 100 μL of diluted plasma was added to 96-well microplates. Plasma FITC concentration was measured by the fluorescence intensity (excitation, 492 nm; emission, 525 nm) with a microplate reader (Synergy H1, Biotek).

### 2.11 Cohousing experiment

After weaning, age- and sex-matched WT and IKO mice were allocated to 4 groups, the WT (10 mice) and IKO (8 mice) groups, which were housed separately, and the WT-co (9 mice) and IKO-co (9 mice) groups, which were housed together (each cage had the same number of mice from each genotype). The mice were on chow diet until they reached 8 weeks old, and then fed an HFD for 15 weeks. Weekly body weight was recorded. After 10 and 11 weeks of HFD feeding, the OGTT and ITT were carried out, respectively. The plasma lipid and insulin levels were measured using commercial kits described above. Kits from BioSino Bio-Technology and Science Incorporation were used to measure the levels of HDL-c and LDL-c.

### 2.12 16S rRNA sequencing experiment

For 16S rRNA sequencing, feces were freshly collected into sterile tubes, stored in an −80 °C fridge, and extracted by the CTAB method. Distinct regions in 16S rRNA genes were amplified with barcoded specific primers. Phusion High-Fidelity PCR Master Mix (New England Biolabs) was used in all PCRs, and PCR products were then validated and quantified. Sequencing libraries were generated using a TruSeq DNA PCR-Free Sample Preparation Kit (Illumina, USA). After adding index codes, the library was sequenced on an Illumina NovaSeq platform to generate paired-end reads with a length of 250 bp. Reads were allocated to samples depending on the unique barcode and then merged to generate raw tags, which were then filtered to get high-quality clean tags based on the QIIME (V1.9.1) quality-controlled process. Using the UCHIME algorithm, the clean tags were compared with the reference database (Silva database) to obtain the effective tags. OTUs were produced by Uparse software (Uparse v7.0.1001), and the cut-off of similarity for assigning sequences to the same OTUs was 97%. Further taxonomic annotation of OTUs was based on a representative sequence generated by the Silva database. After normalization, alpha diversity was used to analyze the diversity within the species through Shannon indices. Beta diversity analysis was performed with QIIME software using weighted UniFrac distances. Principal coordinate analysis (PCoA) was performed *via* the WGCNA, stat and ggplot2 packages in R software (version 2.15.3). LEfSe analysis was used to identify significantly changed microbiomes based on relative abundance, and the LDA score threshold was set to 4.

### 2.13 RNA sequencing experiment

Total RNA from the ileum mucosa of WT and IKO mice was extracted using a standard protocol, and its quality was assessed. 1 μg RNA per sample was used to generate sequencing libraries using the NEBNext® Ultra™ RNA Library Prep Kit for Illumina® (NEB, USA). To assign sequences to samples, index codes were added. Samples with index codes were clustered on flow cells on a cBot Cluster Generation System using the TruSeq PE Cluster Kit v3-cBot-HS (Illumina). Then, the prepared libraries were sequenced on an Illumina NovaSeq platform to generate 150-bp paired-end reads. The in-house Perl scripts were used to process raw reads, and high-quality clean data were obtained. Alignment of paired-end clean reads with the reference genome was performed by HISAT2 v2.0.5, and mapping of reads to genes was counted by the R package featureCounts v1.5.0-p3. The obtained read counts were then analyzed by the DESeq2 R package to obtain normalized gene counts and differentially expressed genes. Genes with an adjusted *P* value < 0.05 were considered differentially expressed, and *P* values were adjusted using Benjamini and Hochberg's method to control the false discovery rate. The GSEA tool (http://www.gsea-msigdb.org/gsea/index.jsp) developed by the Broad Institute was used to analyze the potential enrichment of interesting gene sets according to the guidelines. The hallmark and C2 collections of the Molecular Signatures Database (MSigDB) were used. The C2 collections of KEGG pathways were customized with the addition of previously reported LXR targets as a new gene set.

### 2.14 Analysis of bile acids

Frozen, dried feces, ileum mucosa and plasma were thawed at 4 °C. For feces and tissues, approximately 10 mg were weighed; for plasma, 50 μL was used. After the addition of 100 μL of ddH_2_O, the feces and tissues were homogenized with a homogenizer. The internal standard chlorpropamide (Sigma–Aldrich, Cat# C1290) was added, and the mixture was vortexed, centrifuged at 4 °C and 16,000×*g* for 10 min. 5 μL supernatants were collected and injected. BA standards (Sigma, USA) were dissolved in a water-acetonitrile-isopropanol mixture (10:6:5) to obtain a 10 mg/mL solution. The BA concentration in the supernatants was determined using an Ekspert Ultra LC100 and AB SCIEX Triple TOF 5600 (Waters Corp., Milford, MA, USA) system. An XBridge Peptide BEH C18 column (2.1 mm × 100 mm, 3.5 μm, Waters Corp.) was used to perform chromatographic separation with the following settings: column temperature set to 40 °C, flow rate set to 0.4 mL/min, a mixture of 0.1% formic acid in water and acetonitrile was used as the mobile phase, gradient elution and negative mode were applied. The acquired mass range is *m*/*z* 50–800. With the BA standard database as a reference, different BA metabolites in the samples were quantified by PeakView 1.2 software and MultiQuant 2.1. The concentrations of all BAs in the samples were obtained after correction with an internal standard.

### 2.15 Analysis of ketone bodies

The plasma and ileal *β*-hydroxybutyrate and acetoacetate levels were measured alongside the tricarboxylic cycle carboxylic acids (see below). The fasting plasma *β*-hydroxybutyrate concentration was measured using kits from Sigma–Aldrich (MAK041).

### 2.16 Measurements of succinate and TCA metabolites

The levels of the tricarboxylic cycle carboxylic acid intermediates were measured as described^13^. Briefly, solution A (20 μL) and solution B (50 μL) are mixed freshly and 5 μL of carboxylic acid standard solution or plasma sample was added. Then the resulting mixture was vortexed for 30 s, incubated at 70 °C for 20 min and centrifuged for 10 min at 4 °C and 14,000 rpm (5430R, Eppendorf, Hamburg, Germany). 5 μL of the supernatant solution was collected and used for LC−MS/MS analysis. The ileum and brown adipose tissue were thawed on ice, tissue weight was also recorded. Then, 400 μL of methanol was added, and the tissues were homogenized on ice. The homogenates were mixed by ultrasonication for 3 min on ice and centrifuged at 20,000 × *g* for 10 min at 4 °C. The supernatants were collected, and the following steps were the same as those for standards and plasma samples.

### 2.17 Sodium succinate treatment

WT and IKO mice were fed with HFD for 24 weeks and then treated with disodium succinate (HY-W015410, MedChemExpress) dissolved in drinking water. Their water consumption was monitored, and the water bottles were replaced every 3 days. Mice were weighed weekly. OGTTs and indirect calorimetry were conducted during a 2.5% sodium succinate administration period.

### 2.18 BAT ROS measurement

WT and IKO mice fed with HFD for 5 weeks were fasted overnight and refed for 5 h. The mice were sacrificed, and BAT was separated. ROS levels in BAT were measured by kits from Bestbio (BB-470538, Shanghai, China) following the manufacturer's instructions.

### 2.19 RNA extraction and quantitative reverse transcription PCR analysis

The mouse liver, pancreas, epididymal adipose tissue, brown adipose tissue, muscle, kidney, and the duodenum, jejunum, ileum, and colon mucosa were separated, snap frozen in liquid nitrogen and then transferred to a −80 °C refrigerator. Total RNA was extracted with TRIzol reagent (Invitrogen, Cat# 15596018) was used following the manufacturer's instructions. 2 μg of total RNA to synthesize cDNA by High-Capacity cDNA Reverse Transcription Kit (Applied Biosystems, Cat# 4368813) with random primers. SYBR Green qPCR Master Mix (Bimake, B21203) was used, and the total volume is 20 μL. PCR was performed using QuantStudio 3 Real-Time PCR system (Applied Biosystems). The primers used are listed in Supporting Information Table S1. The relative expression of genes was normalized to that of *36b4/Rplp0* mRNA.

### 2.20 Western blot experiments

Liver and intestine tissue were homogenized using RIPA buffer (Beyotime, P0013B) with the addition of protease and phosphatase inhibitors. Total proteins were quantified by BCA protein assay, and equal amounts were loaded onto gels and separated by SDS‒PAGE. Separated proteins were transferred to PVDF membranes. After blocking with 5% nonfat milk in Tris-buffered saline (TBS), the immunoblots were incubated with the indicated primary antibodies overnight at 4 °C. The blots were washed with TBST for 3 times and incubated with horseradish peroxidase-conjugated secondary antibodies for 1 h at room temperature. The membranes were washed in TBST again at least three times, and then the Amersham Imager 600 (GE Healthcare Life Sciences) was used to detect the protein bands. The antibodies used were antibodies against *β*-actin (Proteintech, Catalog# 66009-1-Ig), ABCG5 (Proteintech, Catalog# 27722-1-AP), ABCG8 (Abcam, Catalogue# ab223056), ACLY (Cell Signaling Technology, Catalog# 4332), FASN (Cell Signaling Technology, Catalog# 3180), SCD1 (Cell Signaling Technology, Catalogue# 2794), CD36 (Santa Cruz, Catalog# sc-7309), FABP1 (Santa Cruz, Catalog# sc-271591), and HSP90 (Proteintech, Catalog# 60318-1-Ig).

### 2.21 Mouse intestinal epithelial cell isolation and organoid culture

The isolation of intestinal epithelial cells and subsequent organoid culture were performed as described previously^14^. Briefly, WT mice were anesthetized by injecting avertin intraperitoneally. The small intestine was cut longitudinally, washed with cold PBS, and cut into pieces smaller than 5-mm. Tissue pieces were then washed three times with crypt chelating buffer (5 mmol/L EDTA, 0.5 mmol/L DTT, 43.4 mmol/L sucrose and 54.9 mmol/L D-sorbitol in PBS, sterilized with a 0.22-μm filter) on an orbital shaker. The obtained fragments were washed again with dissociation buffer (43.4 mmol/L sucrose and 54.9 mmol/L D-sorbitol in PBS, sterilized with a 0.22-μm filter) by vigorous shaking. The villus fraction in supernatant was removed by a 70-μm filter, and the crypt fraction was collected. The isolated crypts were centrifuged at 150 × *g*, 4 °C and resuspended in ice-cold PBS. The crypts were then embedded in Matrigel (BD Biosciences) and seeded on a pre-heated 24-well plate (about 500 crypts per well). The plate was then put back to the incubator for 30 min to allow polymerization. Matrigel was then overlaid with advanced DMED/F12 medium supplemented with penicillin/streptomycin, N2, B27, *N*-acetylcysteine, EGF (50 ng/mL, Invitrogen), noggin (100 ng/mL, R&D) and R-spondin1 (500 ng/mL, R&D). For LXR and PPAR*α* agonist treatment, the organoids were treated with GW3965 (1 μmol/L) or Wy14643 (5 μmol/L) for 12 h. After treatment, the medium was discarded, and cold PBS was added. After centrifugation, the pellets were collected and stored at −80 °C.

### 2.22 Data analysis and availability

All experimental data are expressed as the means ± standard error of mean (SEM). The replicates are biologically independent samples. The sample size was designed based on past experiments and the published studies that utilized similar experimental procedures. At least 5 mice per group were included and, 3 biological replicates were performed for cell experiments. For information about key resources used in this study, see Supporting Information Table S2. The data were analyzed using GraphPad Prism 7.0 (GraphPad Software, San Diego, CA, USA). Unpaired two-tailed Student's *t* tests were used for comparisons between two groups, and comparisons among three groups were performed by one-way ANOVA. Analysis of the data from the cohousing experiment was performed by two-way ANOVA. Statistical analysis of the 16S rRNA and bulk RNA sequencing data was conducted as described in the corresponding methods. No data were excluded. Unless stated otherwise, the researchers who participated in the present study were not fully blinded throughout the sample collection and analysis processes. Raw data from 16S rRNA sequencing and RNA sequencing have been deposited to the SRA (NCBI Sequence Read Archive) and GEO (Gene Expression Omnibus) databases with the accession numbers PRJNA995891 and GSE237833, respectively, which will be publicly available by the date of publication.

## 3 Results

### 3.1 Conditional NCoR1 knockout in IECs ameliorated obesity and metabolic syndrome in mice with diet-induced obesity

**Figure 1:**
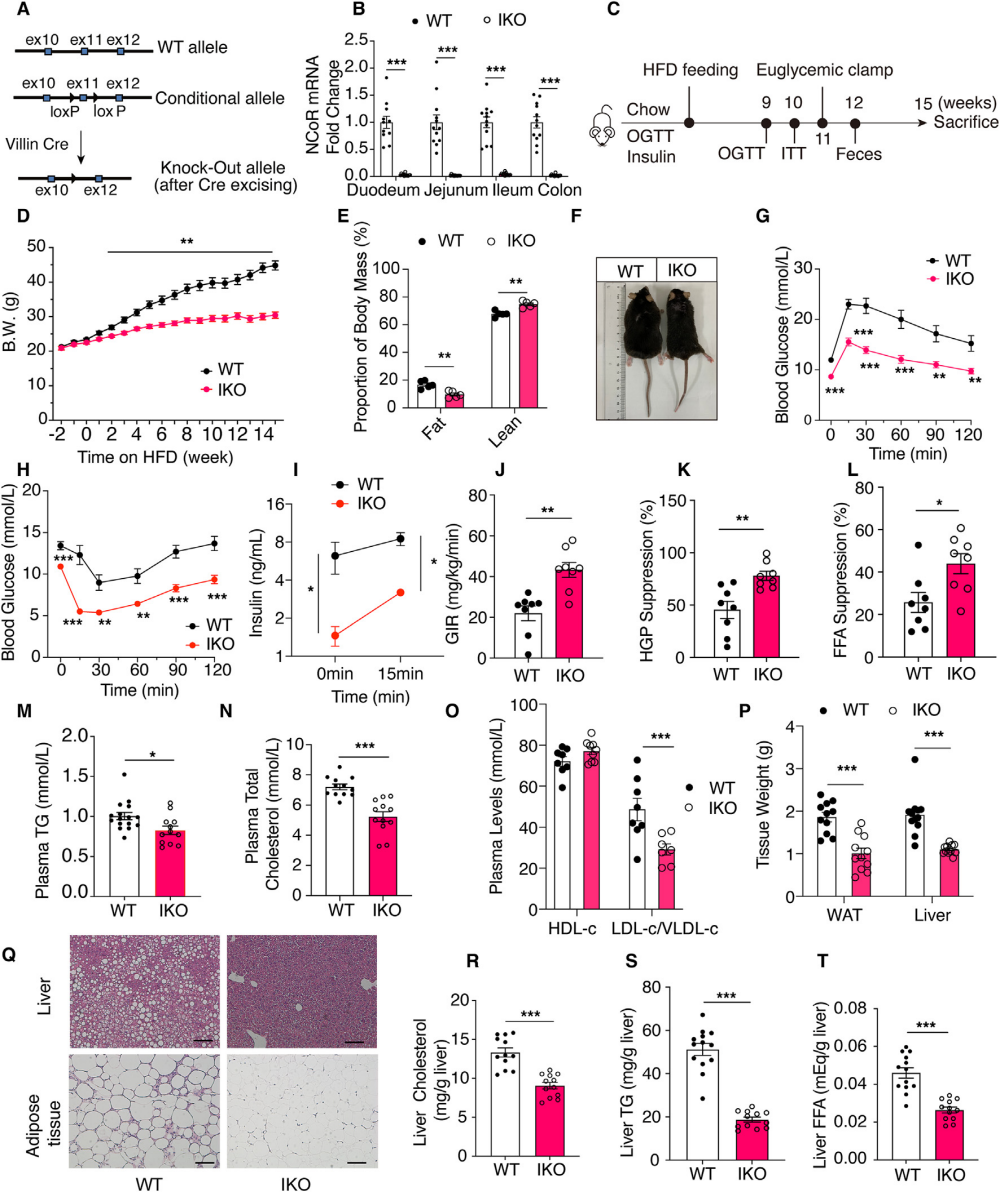
Conditional NCoR1 knockout in IECs greatly ameliorated obesity and metabolic syndrome in mice with diet-induced obesity. (A) WT allele, conditional floxed allele, and deleted NCoR1 gene loci are shown. (B) NCoR1 mRNA expression in different parts of the intestine of WT and IEC-specific NCoR1-knockout mice (*n* = 12). (C) Schematic representation of the experimental design. Nine-week-old WT and IKO mice were used. Chow diet feeding lasted for 3 weeks before the diet was switched to HFD. Phenotypic studies were performed at the designated time points. (D) Weekly growth curves of WT and IKO mice fed with HFD (*n* = 12). (E) Body fat mass and lean mass ratio of mice with 5 weeks of HFD feeding (*n* = 5). (F) Representative photos of mice fed with 9 weeks of HFD. (G) Oral glucose tolerance test, 2 g/kg BW of glucose solution used (*n* = 12). (H) Insulin (0.3 U/kg BW) tolerance test (*n* = 12). (I) Plasma insulin levels of HFD-fed WT and IKO mice in the fasting state and 15 min after oral glucose loading (*n* = 12). (J–L) Hyperinsulinemic-euglycemic clamp experiment. (J) The glucose infusion rate reflects the systemic insulin sensitivity. (K) The suppression of hepatic glucose production represents the insulin sensitivity of the liver. (L) FFA suppression reflects the insulin sensitivity of adipose tissue (*n* = 8). (M) Plasma TG levels after 6 h of fasting (*n* = 12). (N) Plasma TC levels after 6 h of fasting (*n* = 12). (O) Plasma HDL-c and LDL-c/VLDL-c levels after 6 h of fasting (*n* = 8–9). (P) Tissue weight of WAT and liver (*n* = 11–12). (Q) H&E staining of liver and WAT. Representative images are shown. Scale bar = 100 μm. (R–T) Hepatic TC (R), TG (S) and FFA (T) levels (*n* = 12). Experimental data are expressed as the means ± SEM. Two-tailed unpaired Student's *t* test was used for statistical analysis. Statistical significance is expressed as ∗*P* < 0.05, ∗∗*P* < 0.01, ∗∗∗*P* < 0.001.

To assess whether intestinal NCoR1 could regulate metabolic syndrome, we generated conditional IEC NCoR1-knockout (IKO) mice utilizing the Cre-LoxP method (Fig. 1A). Quantitative reverse transcription PCR (RT-qPCR) found that approximately 90% of *NCoR1* was deleted in IECs (Fig. 1B) and that the *NCoR1* levels in other tissues, such as the liver, muscle, brown adipose tissue (BAT), white adipose tissue (WAT) and pancreas (Supporting Information Fig. S1A), were not changed. We also observed no significant changes in the morphology of the small intestine (Fig. S1B).

IKO mice and their wild-type (WT) littermates displayed no differences in body weight, glucose tolerance, or plasma insulin levels (Fig. S1C–S1E) when consuming a standard chow diet. In contrast, when these mice were challenged with a high fat diet (HFD) (Fig. 1C), the IKO mice gained much less weight than their WT counterparts (Fig. 1D). Only the fat mass but not lean mass of IKO mice was significantly lower as shown by body composition measurements (Fig. 1E, Fig. S1F). NCoR1 deletion did not affect mouse development because chow diet-fed WT and IKO mice displayed indistinguishable body weights (Fig. S1C), and the body length of the two groups was also similar (Fig. 1F). Compared with the WT mice, glucose intolerance, insulin resistance and hyperinsulinemia were all greatly improved in the IKO mice (Fig. 1G–I, Fig. S1G and S1H), indicating that NCoR1 deficiency in IECs results in an insulin-sensitive phenotype. The hyperinsulinemic euglycemic clamp further confirmed the insulin-sensitive phenotype in HFD fed IKO mice because both the glucose infusion rate and the glucose disposal rate were significantly increased in the IKO mice (Fig. 1J, Fig. S1I). More specifically, liver and adipose tissue insulin sensitivity were improved in the IKO mice because suppression of both hepatic glucose production and adipose lipolysis (the plasma free FA (FFA) levels) were higher in IKO mice (Fig. 1K and L, Fig. S1J). In line with the lean phenotype, the plasma triglyceride (TG) and total cholesterol (TC) levels were reduced by 26% and 30%, respectively, in HFD fed IKO mice (Fig. 1M and N). The reduction in the TC levels was caused by decreases in the low-density lipoprotein cholesterol (LDL-c) and very low-density lipoprotein cholesterol (VLDL-c) levels (decreased by 45.2%) because the high-density lipoprotein cholesterol (HDL-c) levels remained unchanged (Fig. 1O). Consistently, the IKO mice exhibited improvements in liver appearance and adiposity (Fig. S1K). The tissue weight of the liver and WAT and the tissue weight/body weight ratios were also significantly lower in the IKO mice (Fig. 1P, Fig. S1L). Similarly, H&E staining revealed marked reductions in the liver lipid content and adipocyte size (Fig. 1Q). Compared with those in the WT mice, the hepatic TC, TG, and FFA levels in the IKO mice were decreased by 32%, 64.7%, and 44.4%, respectively (Fig. 1R–T). Together, these results showed that NCoR1 deficiency in IECs markedly attenuated obesity and metabolic syndrome in mice with diet-induced obesity.

### 3.2 Intestinal NCoR1 deficiency increased energy expenditure

**Figure 2:**
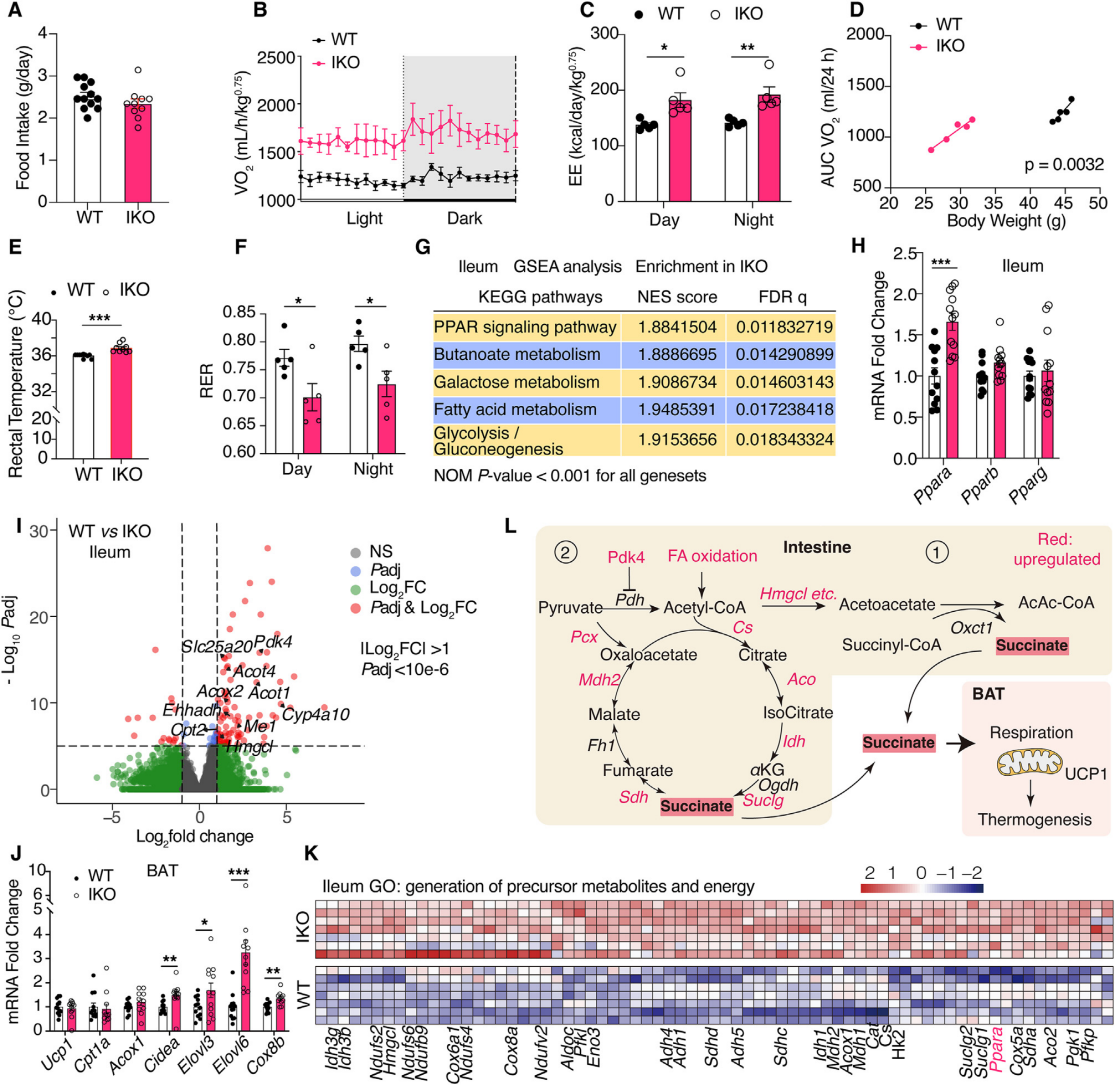
NCoR1 deficiency in IECs increased the energy expenditure and intestinal PPAR*α* signaling. (A) Daily food intake on the 8th week of HFD feeding (*n* = 12). (B–D) Energy expenditure was monitored using a Columbus CLAMS at 12 weeks of HFD feeding. (B) Whole-body oxygen consumption rate. (C) Energy expenditure (heat production). (D) Area under the curve (AUC) of oxygen consumption versus the body weight. The *P* value shown was calculated by nonlinear regression analysis with a straight-line model, which is equivalent to analysis of covariance (ANCOVA). (E) Rectal temperatures of another cohort of HFD-fed (for 8 weeks) WT and IKO mice (*n* = 9). (F) Respiratory exchange ratios (*n* = 5 per group). (G) Top 5 enriched pathways identified by GSEA of RNA sequencing data utilizing KEGG gene sets (*n* = 7). (H) RT-qPCR measurements of three PPAR isotypes in the ileum of HFD-fed WT and IKO mice (*n* = 12). (I) Volcano plot of differentially expressed genes in RNA sequencing data. Target genes of PPAR*α* were labeled (*n* = 7). (J) RT-qPCR measurements of *Ucp1* and FA metabolism pathway genes in the BAT of HFD-fed WT and IKO mice (*n* = 12). (K) Heatmap of genes involved in the “generation of precursor metabolites and energy” pathway from GO analysis (*n* = 7). (L) Schematic illustration of the proposed mechanism of the ileal succinate production by PPAR*α* signaling pathway. Experimental data are expressed as the mean ± SEM. Data are analyzed by unpaired two-tailed Student's *t* test, the R package DESeq2 or GSEA software. Statistical significance is shown as ∗*P* < 0.05, ∗∗*P* < 0.01, ∗∗∗*P* < 0.001.

Given that remarkable improvements were observed in body weight and lipid profiles, we measured the balance between energy expenditure and nutrient intake. The recorded food intake did not differ between WT and IKO mice during either the first week, when normal chow was switched to HFD (Supporting Information Fig. S2A), or during the eighth week of HFD feeding (Fig. 2A). The cumulative food intake throughout the HFD feeding period was also unaffected (Fig. S2B and S2C). Therefore, NCoR1 deficiency in IECs did not inhibit the appetite of the mice. We then measured the energy expenditure by placing the HFD-fed mice under a CLAMS indirect calorimeter monitoring system. Compared with the WT mice, the IKO mice showed significantly higher energy expenditure, as demonstrated by the energy expenditure rate, average oxygen consumption, and AUC of oxygen consumption after correction for the body weight (Fig. 2B–D, Fig. S2H). Consistently, the rectal temperatures were also greater in the IKO mice compared with the WT mice (Fig. 2E), indicating improved thermogenesis in the HFD-fed IKO mice. The respiratory exchange ratio (RER) decreased to approximately 0.7 in IKO mice, reflecting a switch in the body's major energy source from carbohydrates to FAs (Fig. 2F). No difference in locomotor activity during the dark and light cycles was found between the WT and IKO mice (Fig. S2D). Collectively, these results indicated that intestinal NCoR1 deficiency stimulated diet-induced thermogenesis and increased energy expenditure.

### 3.3 Intestinal NCoR1 deficiency upregulated PPARα signaling and promoted succinate-mediated thermogenesis

To understand how intestinal NCoR1 deficiency increased thermogenesis, we assessed the changes in the ileal transcriptome of HFD-fed WT and IKO mice by RNA sequencing. Gene set enrichment analysis (GSEA) using Kyoto Encyclopedia of Genes and Genomes (KEGG) gene sets revealed that the PPAR signaling pathway and its downstream pathways, such as butanoate metabolism (ketogenesis) and FA metabolism (mitochondrial and peroxisomal oxidation), were among the top 5 enriched pathways in the IKO mice (Fig. 2G and Fig. S2E). RT-qPCR analysis found that of the three PPAR isoforms, only *Ppara* was significantly upregulated in IKO mice (Fig. 2H). Supporting this, several target genes of PPAR*α* like *Cyp4a10*, *Pdk4*, *Acot2*, *Hmgcl*, *Acox1* and *Acaa1b* were significantly upregulated in IKO mice measured by RNA seq and RT-qPCR (Fig. 2I and Fig. S2F). Therefore, these results indicate that PPAR*α* was the major NR activated by NCoR1 deficiency in the intestine under obesity conditions.

PPAR*α* agonist treatment in mice reduced body weight and increased thermogenic gene expression in BAT^6^. So, we measured changes in thermogenic tissues to see whether intestinal PPAR*α* activation had similar effects. In BAT, the expression of thermogenic genes, including *Ucp1, Pgc1a*, *Dio2,* and *Prdm16*, did not differ between the groups (Fig. 2J and Fig. S2G). Similarly, the expression of browning markers, including *Ucp1, Tmem26 and Cidea*, in WAT (Fig. S2I) were also unchanged. Thus, intestinal PPAR*α* activation didn't increase BAT thermogenesis through transcriptional control. BAT thermogenesis could also be induced by increased levels of free FAs, which activated PPAR*α* signaling, supplied acetyl-CoA to the tricarboxylic cycle and increased mitochondrial oxidative phosphorylation to stimulate uncoupling^15^. However, lipidomic analysis revealed that the abundance of total FAs and long-chain FAs in BAT only showed an increasing trend (Fig. S2J and S2K). Moreover, the expression of *Cpt1a* and *Acox1*, which encoding two rate-limiting enzymes in FA oxidation, remained unchanged (Fig. 2J). Instead, genes involved in FA elongation (*Elovl3* and *Elvol6*) and lipid droplet formation (*Cidea*)^16^ pathways were significantly upregulated (Fig. 2J). Plasma FFA levels were also unchanged by intestinal NCoR1 deficiency (Fig. S2L). Thus, FAs and FA oxidation may not directly affect BAT thermogenesis in IKO mice. Notably, *Elovl3* and *Elvol6* are also important for the adaptation of BAT when exposed to cold. When stimulated, BAT upregulates *Elovl6* to promote the expression of mitochondrial respiratory electron transport chain (ETC) components^17^. Consistently, *Cox8b*, a component of the ETC complex IV, was significantly upregulated in the IKO mice. Taken together, these data suggested that the increase in energy expenditure in the IKO mice may be mediated by certain gut-derived metabolite that can directly increase mitochondrial respiration in BAT.

In the intestine, Gene Ontology (GO) enrichment analysis of the differentially expressed genes (DEGs) showed that “generation of precursor metabolites and energy” pathway was enriched of IKO mice (Fig. S2M). The DEGs in this pathway consisted of genes in PPAR*α* regulated signaling pathways and genes in the downstream tricarboxylic cycle (*Idh3*, *Sdha*, *Sdhc*, *Suclg2*, *Mdh2*, *Cs*, *Aco2*, etc.) and oxidative phosphorylation (*Ndufs2*, *Ndufs6*, *Cox6a1*, *Cox8a*, etc.) pathways (Fig. 2K). The intermediates of the tricarboxylic cycle can be transported out of mitochondria and participate in cellular processes in other tissues. Particularly, the intermediate succinate, which can participate in respiration through the ETC complex II member succinate dehydrogenase, was able to stimulate BAT respiration and thermogenesis, whereas other intermediates couldn't^18^. Our RNA seq data suggested that the production of succinate could be stimulated by PPAR*α* activation *via* two proven mechanisms (Fig. 2L). In mechanism 1, enhanced ketogenesis resulted in the synthesis of more acetoacetate, which is subsequently metabolized to acetoacetyl-CoA; at the same time, succinyl-CoA is converted to succinate^19^. In mechanism 2, *Pdk4* (a target gene of PPAR*α* whose expression was increased 10-fold in IKO mice) is a natural inhibitor of pyruvate dehydrogenase (PDH). Inhibition of PDH increased the flux of pyruvate metabolism to the tricarboxylic cycle *via* pyruvate carboxylase (*Pcx*; its expression was increased twofold in IKO mice). Together with increased acetyl-CoA from the FA oxidation pathway, these changes led to the accumulation of succinate through the tricarboxylic cycle^20^^,^^21^.

**Figure 3:**
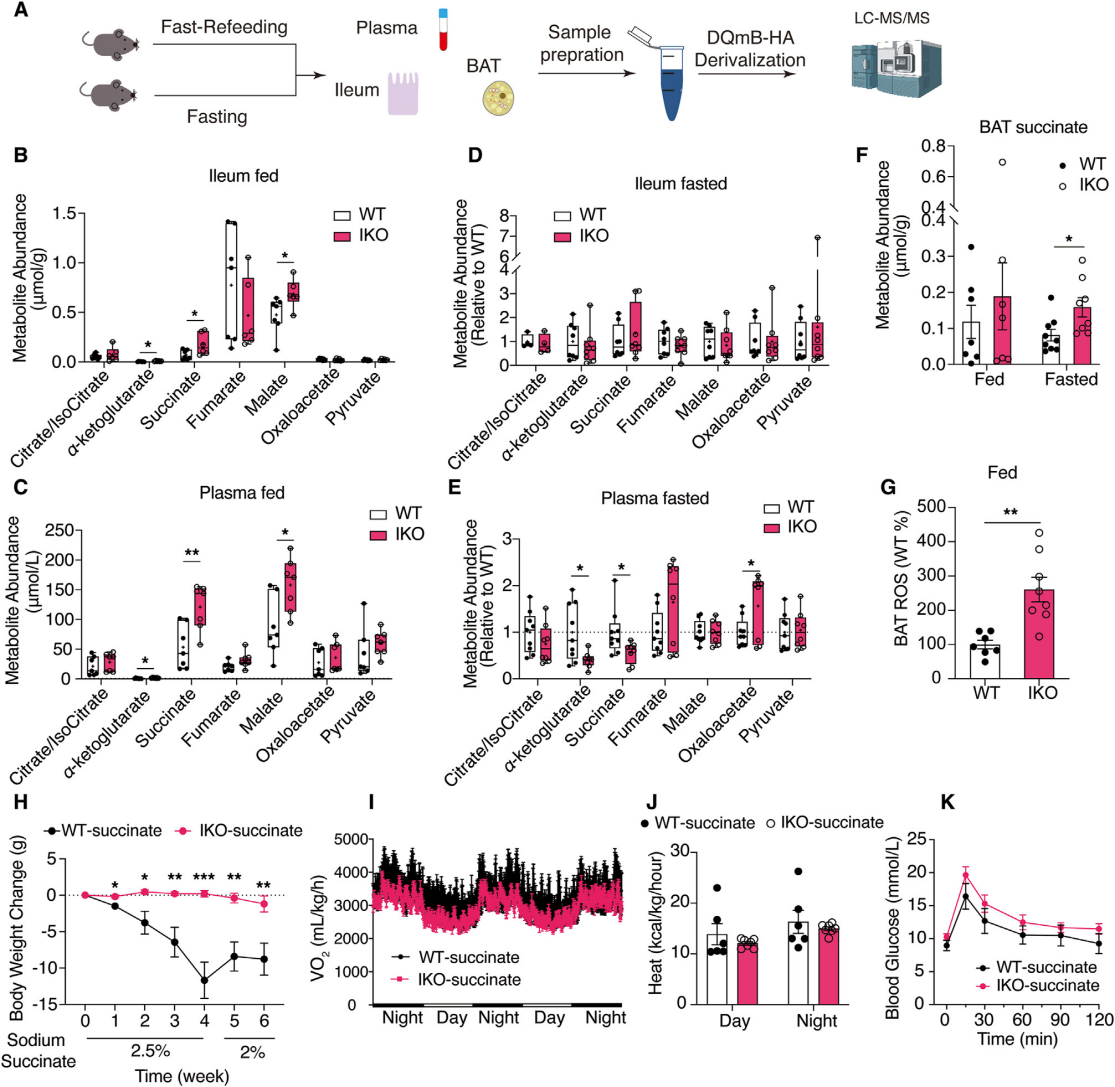
Intestinal NCoR1 deficiency increased postprandial succinate production and promoted succinate-mediated thermogenesis. (A) Schematic illustration showing procedures of measuring the levels of metabolites associated with ileal PPAR*α* activation by LC‒MS/MS in the ileum, plasma, and BAT of WT and IKO mice fed with HFD. (B) Concentrations of the tricarboxylic acid cycle intermediates in the ileum after 6 h of refeeding (*n* = 6–7). (C) Plasma levels of the tricarboxylic acid cycle intermediates after 6 h of refeeding (*n* = 7). (D) Concentrations of the tricarboxylic acid cycle intermediates in the ileum after 5 h of fasting (*n* = 8). (E) Plasma levels of the tricarboxylic acid cycle intermediates after 5 h of fasting (*n* = 8–9). (F) Relative abundance of succinate in the BAT at the fed and fasted state (*n* = 7–9). (G) ROS levels in BAT after 5 h refed (*n* = 7–8). (H) Changes in body weight after the administration of sodium succinate to WT and IKO mice (fed with HFD for 24 week) (*n* = 8). (I, J) Oxygen consumption rate (I) and (J) energy expenditure (heat production) of HFD-fed WT and IKO mice after 4 weeks of sodium succinate administration (2.5%), as measured by CLAMS. (K) Oral glucose tolerance tests (OGTTs) after 4 weeks of 2.5% sodium succinate administration to HFD-fed (24-week-old) WT and IKO mice. Experimental data are expressed as the mean ± SEM. (B–E) The box extends from the 25th to the 75th percentiles with the median shown by the line in the middle, and "+" indicates the mean. Two-tailed unpaired Student's *t* test was used for statistical analysis. Statistical significance was expressed as ∗*P* < 0.05, ∗∗*P* < 0.01, ∗∗∗*P* < 0.001.

Therefore, to validate the RNA sequencing data, the levels of the tricarboxylic cycle intermediates and ketone bodies in the plasma, ileum, and BAT were determined by LC‒MS/MS (Fig. 3A). As expected, the succinate, alpha-ketoglutarate and malate concentrations in refed state were significantly greater in ileum and plasma of the IKO mice. The levels of succinate in the ileum and plasma were increased by 2.55-fold and 2.24-fold, respectively (Fig. 3B and C). Plasma acetyl-CoA levels were also increased in IKO mice (Supporting Information Fig. S3A). In contrast, in the fasted state, the ileal succinate concentration did not change, whereas the plasma succinate level decreased by 43.5%. The levels of other intermediates, such as alpha-ketoglutarate, decreased, but the level of malate did not change (Fig. 3D and E). Among all the intermediates, BAT preferentially sequesters succinate from the circulation^18^. In BAT, the succinate concentration increased significantly by 1.9-fold in the fasted state, with a trend of increase (by 1.6-fold) in the refed state of IKO mice (Fig. 3F). And the BAT ROS levels increased significantly too (Fig. 3G), indicating enhanced thermogenesis in BAT. Moreover, in line with the RNA-seq results, the concentrations of ketone bodies in the ileum and plasma were also increased in the postprandial state (Fig. S3B and S3C), and similar to the results obtained with succinate, when the concentrations of ileal *β*-hydroxybutyrate (*β*-OHB) and acetoacetate (AcAc) did not change, the plasma *β*-OHB levels significantly decreased (Fig. S3B and S3C), probably due to a lack of intestinal production and the downregulation of PPARα signaling in the fasting liver (Fig. S3D and S3E). Together, these results supported our theory that PPAR*α* activation in the intestine produces succinate to increase thermogenesis in BAT.

To further confirm the contribution of succinate to obesity in the IKO mice, we administered 2.5% disodium succinate dissolved in the drinking water to WT and IKO mice (fed with HFD for 24 weeks) for 4 weeks. The WT and IKO mice consumed the same amount of sodium succinate, as revealed by the water consumption (Fig. S3E). Succinate administration significantly decreased the body weight of the WT mice but did not further decrease the body weight of the IKO mice (Fig. 3H). After lowering the concentration of sodium succinate from 2.5% to 2% for 2 weeks, the body weight of the WT mice rebounded, whereas that of the IKO mice remained the same (Fig. 3H). Interestingly, the WT mice consumed more succinate during these two weeks, probably due to the need for higher levels of succinate to support activated BAT thermogenesis (Fig. S3F). The level of oxygen consumption and energy expenditure measured by CLAMS after 2.5% succinate administration were not different between the WT and IKO mice (Fig. 3I and J, Fig. S3G). Consistently, the differences in oral glucose tolerance between groups also diminished (Fig. 3K, Fig. S3H). Collectively, these results showed that IECs with NCoR1 deficiency produced more succinate in the refed state, which contributed to the weight loss and metabolic phenotype of the IKO mice.

### 3.4 The effects of intestinal NCoR1 deficiency were not mediated by the gut microbiota

The gut microbiota has also been shown to play a role in succinate production^22^. To investigate whether NCoR1 deletion in IECs affects the gut microbiome, we conducted a cohousing experiment and evaluated the possible changes and effects by 16S rRNA sequencing. After weaning, the age- and sex-matched WT and IKO mice were divided into four groups (Supporting Information Fig. S4A): the WT and IKO groups, which were housed separately, and the WT-co and IKO-co groups, which were housed together. After chronic HFD feeding, 16S rRNA sequencing was conducted using freshly collected stool. Principal coordinate analysis and Wilcoxon signed-rank tests based on weighted UniFrac distances revealed that NCoR1 deletion in IECs showed no meaningful effect on the gut microbiota overall, regardless of whether the mice were housed separately or cohoused (Fig. S4B–S4D). In both genotypes, the *β* diversity of the cohoused mice significantly differed from that of separately housed mice, as measured by the weighted UniFrac distance (Fig. S4D and S4E). However, such alterations failed to affect most of the metabolic changes induced by NCoR1 deletion in IECs. These metabolic changes, including body weight loss (Fig. S4F), improved glucose tolerance (Fig. S4G), insulin sensitivity (Fig. S4H) and hyperinsulinemia (Fig. S4I) and reductions in the blood TG (Fig. S4J) levels were still significantly different between genotypes regardless of the housing conditions and were comparable between separately housed and cohoused mice of the same genotype. The FFA levels did not change under any conditions (Fig. S4K). In line with the changes in *β*-diversity, cohousing indeed increased the HDL-c levels in both genotypes (Fig. S4L), whereas the LDL-c levels in the same genotype were not significantly affected by cohousing (Fig. S4M). However, because NCoR1 IKO itself could not increase the HDL-c levels and because the LDL-c levels in the IKO and IKO-co groups were comparable, we hypothesized that the changes induced by the gut microbiota did not contribute much to the metabolic improvements observed in the IKO mice. Together, these data demonstrated that the effects of NCoR1 on obesity and metabolic syndrome did not rely on gut microbiota activity.

### 3.5 Intestinal NCoR1 deficiency impaired lipid absorption despite activation of PPARα signaling

**Figure 4:**
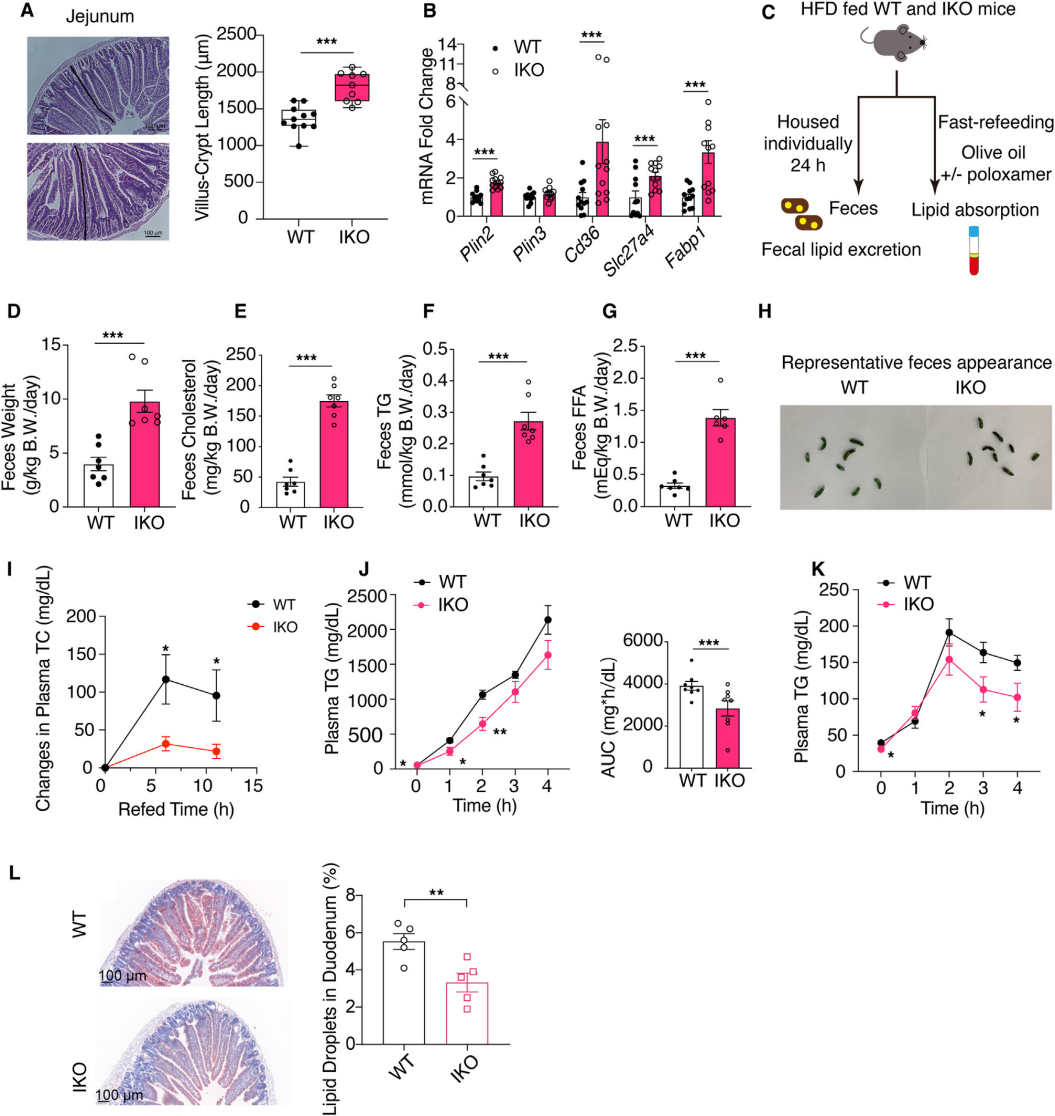
Intestinal NCoR1 deficiency impaired lipid absorption despite PPAR*α* activation. (A) Morphology of intestinal epithelium shown by H&E staining of jejunum tissues from HFD-fed WT and IKO mice. Villus-crypt length was quantified (*n* = 8). (B) RT-qPCR measurements of PPAR*α* target genes responsible for lipid droplet formation and FA uptake in the ileum (*n* = 12). (C) Schematic illustration of the experimental design. The HFD-fed mice were either housed individually for 24 h to collect their feces or subjected to overnight fasting-refeeding and TG tolerance tests to evaluate the changes in lipid absorption. (D) Fecal output of HFD-fed WT and IKO mice over a 24-h period (*n* = 7). (E–G) Fecal TC (E), TG (F), and FFA (G) excretion of HFD-fed WT and IKO mice over a 24-h period (*n* = 7). (H) Representative photographs of feces showing that IKO mice did not produce oily stools. (I) Changes in the basal plasma TC levels in the refed state after overnight fasting (15 h). WT and IKO mice were fed with HFD for 14 weeks (*n* = 8). (J) Plasma TG levels in WT and IKO mice fed with HFD for 13 weeks. Area under the curve (AUC) was also shown. The mice were fasted for 5 h, and poloxamer 407 (1 g/kg BW) was injected intraperitoneally 30 min before being administered with 200 μL olive oil (*n* = 8). (K) Plasma TG levels in WT and IKO mice fed with HFD for 12 weeks. The mice were fasted overnight and administered with 10 mL/kg olive oil (*n* = 5). (L) Oil Red O staining of the duodenum of HFD fed WT and IKO mice (*n* = 8). Experimental data are expressed as the mean ± SEM. Two-tailed unpaired Student's *t* test was used for statistical analysis. Statistical significance is expressed as ∗P < 0.05, ∗∗*P* < 0.01, ∗∗∗*P* < 0.001.

In addition to FA oxidation, PPAR*α* agonism also positively regulates the uptake of FAs. Given that the intestine is responsible for dietary fat absorption, this may hinder the hypolipidemic effects of intestinal PPAR*α* agonism. Previous reports have found that intestinal PPAR*α* deficiency didn't markedly inhibit FA oxidation and ketogenesis (Fig. S5A and S5B), yet it reduced the absorption of dietary fat and improved dyslipidemia and hepatic steatosis^23^^,^^24^. Similarly, in our model, the effects of intestinal PPAR*α* on FA uptake were also observed, as shown by the increased villus-crypt length of the jejunum (Fig. 4A) and the upregulation of FA transporters and lipid droplet formation mediators (*Cd36*, *Slc27a4*, *Fabp1*, and *Plin2*) in the ileum (Fig. 4B). However, NCoR1 IKO mice still exhibited improvements in the circulating TG and cholesterol levels (Fig. 1L–N) and hepatic steatosis (Fig. 1Q–T). Therefore, we next evaluated the effects of intestinal NCoR1 deficiency on lipid absorption (Fig. 4C). Feces excretion and the fecal cholesterol, TG, and FFA levels were increased significantly in the IKO mice (Fig. 4D–G and Fig. S5C). Oily stools were not observed in IKO mice (Fig. 4H), as was the case for orlistat^25^. When the mice were fasted overnight and refed with HFD for 6 h, the increase in the plasma TC and TG levels was significantly lower in the IKO mice, which consumed similar amounts of food (Fig. 4I, Fig. S5D and S5E). Furthermore, intestinal TG absorption measured by oral gavage of olive oil with (Fig. 4J) or without (Fig. 4K) advance treatment with the LPL inhibitor poloxamer-407 showed that NCoR1 deficiency in IECs significantly reduced post-gavage plasma TG levels. Oil Red O staining further confirmed the impaired lipid absorption in the intestine of IKO mice (Fig. 4L). Moreover, intestinal permeability, as shown by FITC-dextran gavage (Fig. S5F), and small intestine length (Fig. S5G) were unaffected. Thus, NCoR1 deficiency in IECs reduced intestinal lipid absorption despite PPAR*α* activation.

### 3.6 Intestinal NCoR1 deficiency derepressed LXR and reduced duodenal cholesterol absorption

**Figure 5:**
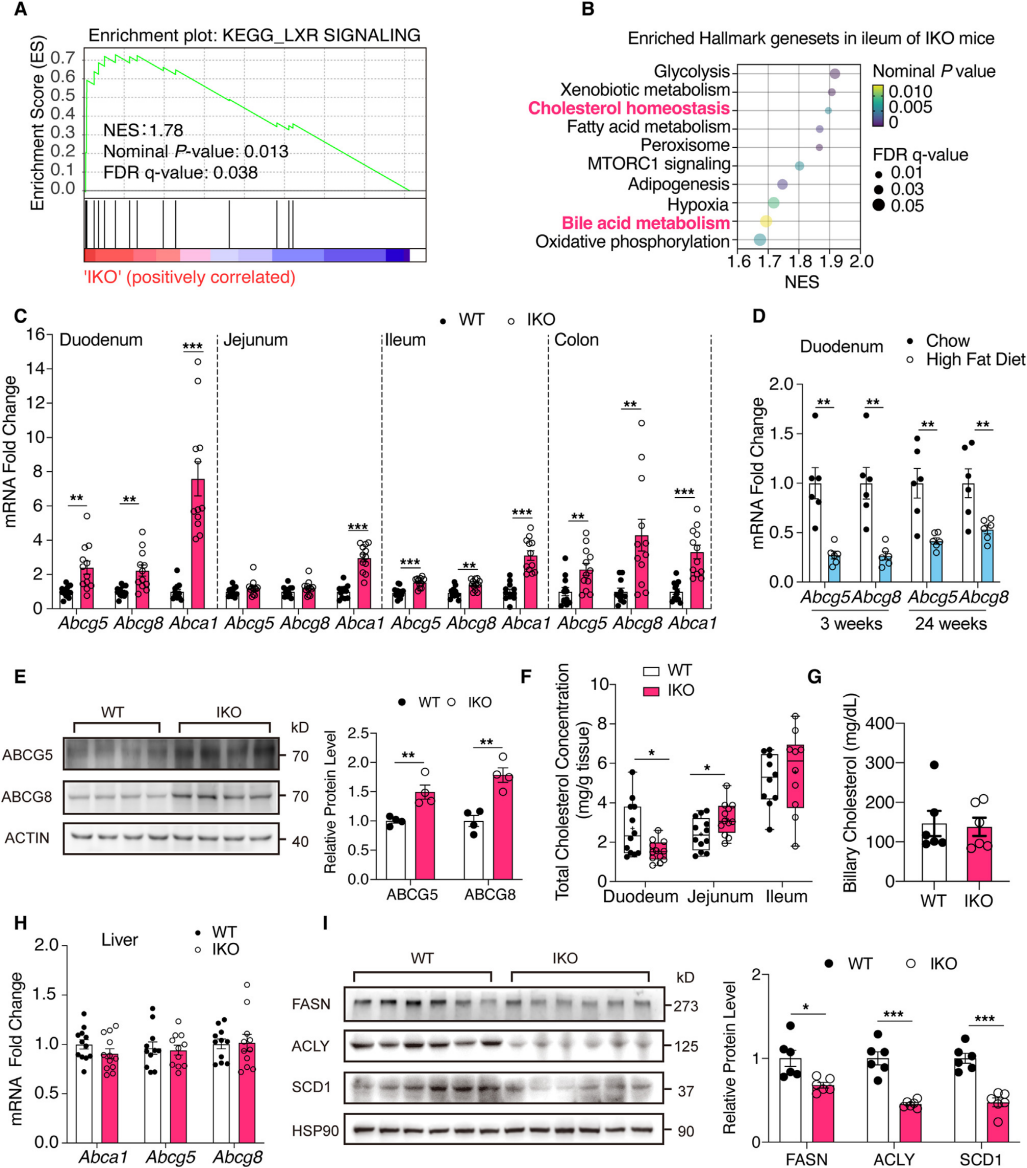
Intestinal NCoR1 deficiency derepressed LXR in the gut and reduced duodenal cholesterol absorption. (A) LXR signaling pathway was enriched in the ileum of HFD-fed IKO mice analyzed by GSEA (*n* = 7). (B) Enriched pathways identified by GSEA of RNA sequencing data utilizing Hallmark gene sets (*n* = 7). (C) RT-qPCR analysis of direct LXR target genes responsible for cholesterol export in the small intestine and colon (*n* = 11–12). (D) mRNA expression of *Abcg5* and *Abcg8* in the duodenum of C57BL/6J mice at 3 weeks and 24 weeks of HFD/chow diet feeding (*n* = 6). (E) Quantification of ABCG5 and ABCG8 protein in ileum of HFD-fed WT and IKO mice by Western blot (*n* = 4). (F) TC concentrations in the duodenum, jejunum, and ileum of HFD-fed WT and IKO mice (*n* = 12) for the duodenum and jejunum (*n* = 10) for the ileum. The box extends from the 25th to the 75th percentiles with the median shown by the line in the middle, and "+" indicates the mean. (G) Biliary cholesterol concentrations of HFD-fed WT and IKO mice (*n* = 6). (H) Hepatic mRNA expression of *Abca1*, *Abcg5*, and *Abcg8* (*n* = 12). (I) Western blot and quantification of lipogenic ACLY, FASN and SCD1 protein expression in the livers (*n* = 6). Experimental data are expressed as the mean ± SEM. (A, B) Statistical analysis was performed with GSEA software. (C–I) Two-tailed unpaired Student's *t* test was used for statistical analysis. Statistical significance was expressed as ∗*P* < 0.05, ∗∗*P* < 0.01, ∗∗∗*P* < 0.001.

The significant reduction in lipid absorption suggested that NCoR1 deletion in IECs may also activate other NRs. Therefore, we examined the RNA-seq data and performed a GSEA using KEGG and Hallmark gene sets, which revealed enrichment in three lipid absorption-related pathways: LXR signaling, cholesterol homeostasis, and BA metabolism (Fig. 5A and B). Because BAs are synthesized from cholesterol, we first focused on cholesterol metabolism. The upregulated cholesterol homeostasis pathway mostly consisted of genes in the cholesterol biosynthesis (mevalonate) pathway and could not explain the observed phenotype (Supporting Information Fig. S6A and S6B). Considering that LXR is the master regulator of cholesterol transport, and that fecal cholesterol was significantly increased, the upregulation of LXR signaling might be the primary reason for the observed decrease in the cholesterol levels. RT‒qPCR analysis of the key LXR target genes *Abcg5*, *Abcg8* (an apical cholesterol exporter^26^) and *Abca1* (a basolateral cholesterol exporter^27^) confirmed LXR activation in all parts of intestine of the IKO mice (Fig. 5C), and these genes were not regulated by the PPAR*α* agonist Wy14643 (Fig. S6C). A comparison of HFD-fed mice with age-matched chow diet-fed mice revealed that duodenal and jejunal LXR signaling was repressed by HFD feeding (Fig. 5D and Fig. S6D); thus, intestinal NCoR1 deletion derepressed LXR in obese mice. Examination of the protein expression of ABCG5 and ABCG8 in the ileum further confirmed the de-repression of LXR (Fig. 5E). The upregulation of *Abcg5* and *Abcg8* promoted cholesterol excretion in feces and reduced fractional cholesterol absorption^28^, whereas *Abca1* upregulation transported more cholesterol in the form of HDL-c into the portal vein^27^, which was consistent with the unchanged HDL-c levels despite a marked reduction in the systemic cholesterol concentrations. Measurement of the TC concentrations in the small intestine revealed that the LXR-induced cholesterol reduction was most significant in the duodenum, whereas the TC concentrations were increased in the jejunum and unchanged in the ileum, probably due to the augmented cholesterol biosynthesis pathway in the lower small intestine (Fig. 5F, Fig. S6A and S6B). The intestinal cholesterol importer *Npc1l1* was also downregulated in the jejunum and ileum (Fig. S6E). However, its expression was decreased by HFD feeding (Fig. S6F), and the cholesterol concentrations in the jejunum and ileum were not reduced; thus, the role of this importer in our model was probably less important.

Restricted LXR activation in the intestine^29^ exhibited robust cholesterol-lowering efficacy and reduces hepatic TG and FA synthesis. Similarly, the de-repression of LXR by NCoR1 deletion was limited to the intestine because the hepatic expression of *Abcg5*, *Abcg8* and *Abca1* remained unchanged (Fig. 5G and H). The biliary cholesterol levels did not change despite the upregulation of hepatic *Serbp2* signaling, which regulates cholesterol biosynthesis (Fig. 5G and Fig. S6G). The protein levels of key components of the hepatic lipogenesis program (ACLY, FASN, and SCD1) were also significantly decreased (Fig. 5I). Taken together, our results demonstrated that in addition to PPAR*α*, NCoR1 deficiency in IECs also derepressed LXR in the intestine, which led to enhanced cholesterol excretion in feces and impaired cholesterol absorption in the duodenum.

### 3.7 Intestinal NCoR1 deficiency altered BA synthesis and reduced duodenal lipid absorption

**Figure 6:**
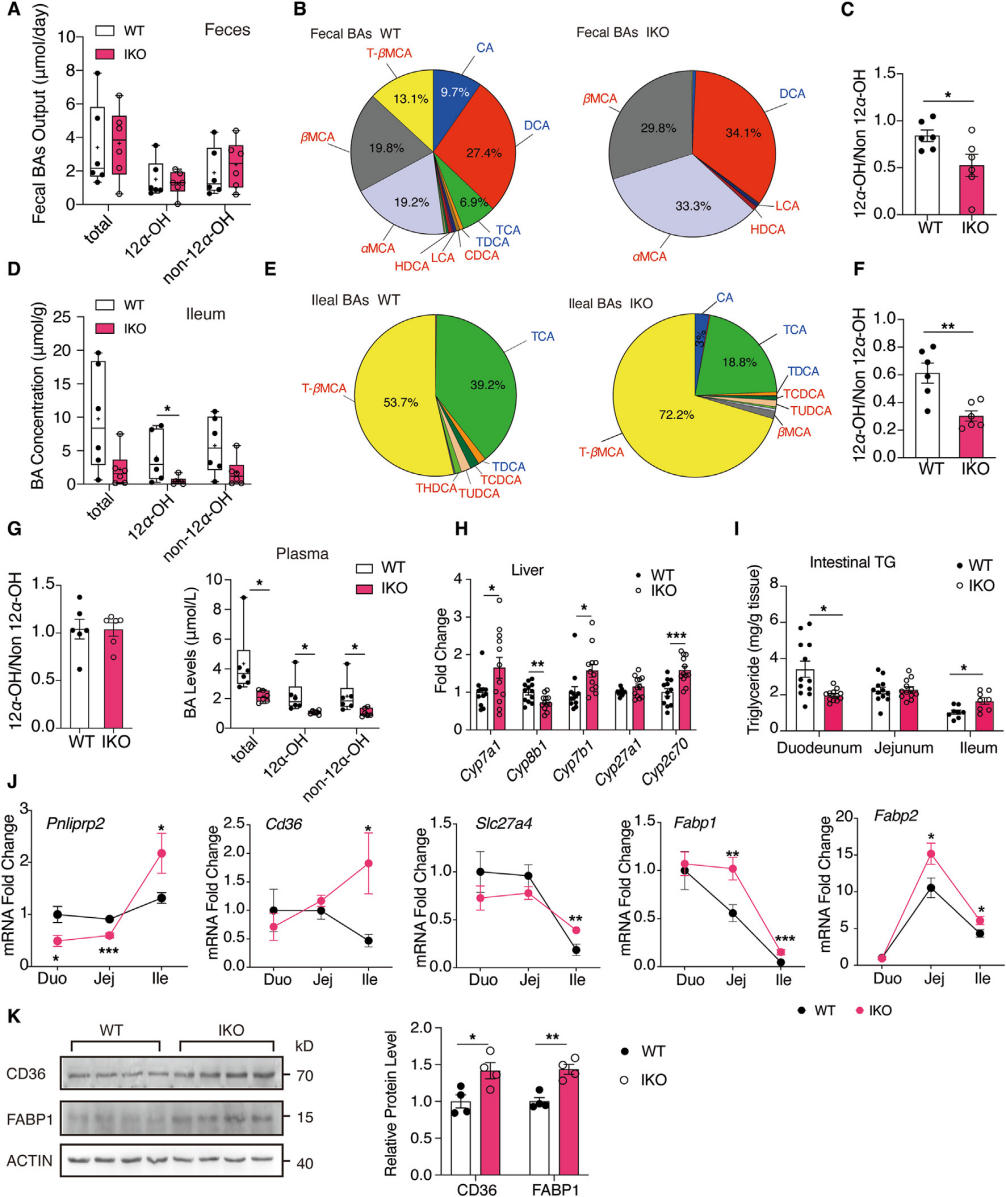
Intestinal NCoR1 deficiency altered BA synthesis and reduced duodenal lipid absorption. (A) Total 24-h fecal output of total, 12*α*-hydroxylated and non-12*α*-hydroxylated BAs in WT and IKO mice fed with HFD for 15 weeks (*n* = 6). (B, C) Fecal BA composition and the ratio of 12*α*-hydroxylated to non-12*α*-hydroxylated BA (*n* = 6). (D) Total, 12*α*-hydroxylated and non-12*α*-hydroxylated BA concentrations in the ileum (*n* = 6). (E, F) Ileal BA composition and the ratio of 12*α*-hydroxylated to non-12*α*-hydroxylated BA ratio (*n* = 6). (G) Total levels of plasma BAs, levels and ratio of 12*α*-hydroxylated and non-12*α*-hydroxylated BAs (*n* = 6). (H) Hepatic mRNA expression of BA synthases (*n* = 12). (I) TG concentrations in the duodenum, jejunum, and ileum (*n* = 12) for the duodenum and jejunum, (*n* = 8) for the ileum. (J) mRNA expression of *Ppara* target genes in TG digestion and FA absorption pathways in the small intestine (*n* = 11–12). (K) Western blot and quantification of CD36 and FABP1 protein in ileum of HFD fed WT and IKO mice (*n* = 4). Experimental data are expressed as the mean ± SEM. (A, D, G) The box spans from the 25th to the 75th percentiles with the median shown by the line in the middle, and "+" indicates the mean. Two-tailed unpaired Student's *t* test was used for statistical analysis. Statistical significance was expressed as ∗*P* < 0.05, ∗∗*P* < 0.01, ∗∗∗*P* < 0.001.

As the source of BA synthesis, reducing the cholesterol levels has been proven to alter the BA levels and composition^30^, ^31^, ^32^. Because BAs are essential for the emulsification and absorption of dietary fat (mainly TGs), we measured the BA profiles in the feces, ileum and plasma. The daily fecal output of BAs was the same between the WT and IKO mice (Fig. 6A), even after correction for the body weight (Supporting Information Fig. S7A). In terms of the BA composition in feces, the quantity of hydrophobic 12*α*-hydroxylated BAs and hydrophilic non-12*α*-hydroxylated BAs was not altered, but the percentages of the latter, mainly muricholic acids (*e*.*g*., *α*-MCA, *β*-MCA and T-*β*MCA), increased, whereas the percentages of the former, mainly cholic acid (CA) and deoxycholic acid (DCA), decreased (Fig. 6B), resulting in a significantly reduced ratio of 12*α*-OH BAs to non-12*α*-OH BAs (hydrophobicity ratio) (Fig. 6C). Similarly, in the ileum, the reduction in 12*α*-OH BAs (mainly taurocholic acid, TCA) led to a significantly decreased hydrophobicity index, whereas the total BA and non-12*α*-OH BA levels showed a nonsignificant decreasing trend (Fig. 6D–F). In plasma, both the hydrophilic and hydrophobic BAs decreased, but not the hydrophobicity index (Fig. 6G). Thus, NCoR1 deficiency in IECs primarily reduced the 12*α*-OH BA levels and reduced the hydrophobicity index in the feces and ileum. The core enriched genes involved in Hallmark BA metabolism geneset participated in BA synthesis (side chain oxidation etc.)^33^, cholesterol transport (*Abcg8*, *Abca1*) and *Fxr*/*Nr1h4* (Fig. S7B). BA synthesis did not occur in the intestine. Previous reports have suggested that reducing the cholesterol levels affects the BA levels and composition partly through feedback regulation of FXR signaling and BA synthetases^31^^,^^34^. Accordingly, RT‒qPCR analysis revealed that the hepatic expression of *Cyp8b1,* which controls 12*α*-OH BA synthesis^35^, was significantly reduced (Fig. 6H), whereas the expression of *Cyp7a1*, *Cyp7b1* and *Cyp2c70*, which contribute to non-12*α*-OH BA synthesis, was increased. The expression of *Fgf15* (key target gene of FXR) was the same between the WT and IKO mice (Fig. S7C) in all parts of the intestine, and other target genes of FXR in the liver remained unchanged (Fig. S7D). Therefore, the effects of NCoR1 deficiency on BA metabolism did not occur through FXR signaling, but rather involved intestinal cholesterol supply and LXR signaling.

Because the BA composition also affects TG and FA absorption, we next investigated the lipid profiles in IECs. In IECs, the TG concentrations were significantly reduced in the duodenum but increased in the ileum (Fig. 6I). The FFA concentrations were also reduced most significantly in the duodenum (Fig. S7E). These results indicated that BAs mainly affected lipid absorption in the duodenum, which was in line with previous reports^35^. At the transcriptional level, the regulation of PPAR*α* on TG digestion (*Pnliprp2*) and FA transport (*Cd36*, *Slc27a4*, *Fabp1* and *Fabp2*) were still evident in the ileum (Fig. 6J). Consistent with qPCR analysis, protein expression of CD36 and FABP1 were significantly increased in the ileum (Fig. 6K). However, the expression of these genes decreased (*i*.*e*., *Pnliprp2*) or remained unchanged in the duodenum (Fig. 6J). Similarly, mRNAs encoding key enzymes involved in TG synthesis in both the glyceraldehyde 3-phosphate pathway (*Gpat4* and *Lpin2*) and the monoacylglycerol pathway (*Mogat2* and *Dgat2*) also exhibited the same pattern (Fig. S7F). Additionally, the *Mttp* gene, which encodes the key TG transfer protein in chylomicron assembly, was upregulated only in the ileum (Fig. S7F). Together, these results demonstrated that although PPAR*α* activation induced lipid absorption in the ileum, the reduction in BA hydrophobicity counteracted these effects and reduced lipid absorption in the duodenum. Because the duodenum is the major absorptive organ, overall lipid absorption was impaired in the IKO mice, and the fecal lipid output was increased.

### 3.8 Induced NCoR1 deletion in IECs had therapeutic effects on obese mice

**Figure 7:**
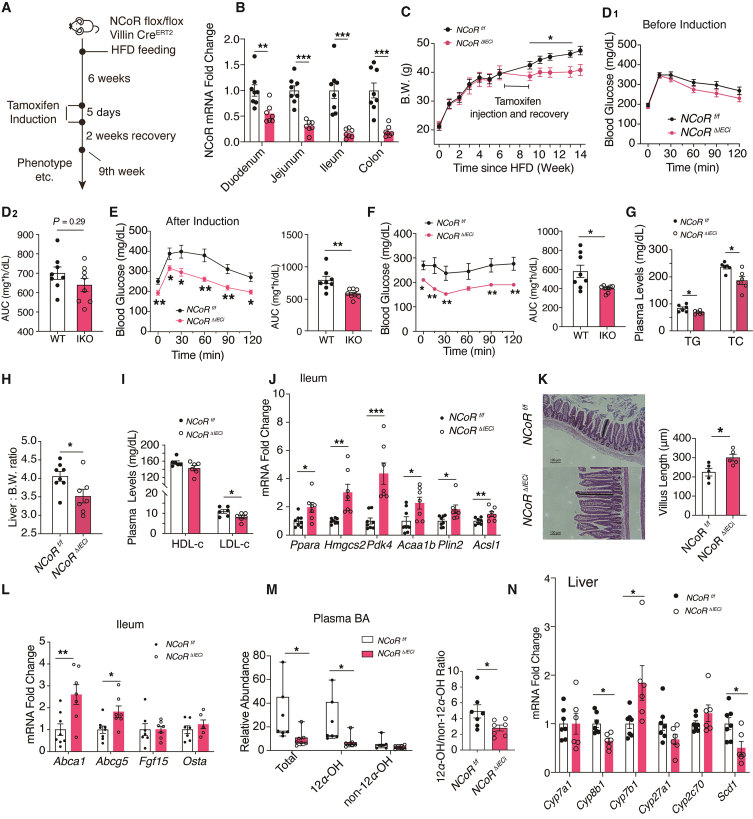
The effects of intestinal NCoR1 deficiency could be elicited after disease onset. (A) Experimental design of the tamoxifen-induced IEC NCoR1 knockout mouse study. Obtained uninduced *NCoR1*^*f/f*^ mice and *NCoR1*^*ΔIECi*^ mice were fed with HFD for six weeks before injecting with tamoxifen for 5 days, which induced deletion of the NCoR1 gene *via* Cre recombinase. HFD feeding continued for another 7 weeks, which included 2 weeks of recovery. OGTTs and ITTs were carried out on the 6th, 10th and 11th weeks of HFD feeding. Other parameters were measured after the mice were sacrificed. (B) RT-qPCR analysis of NCoR1 in the intestines of *NCoR1*^*f/f*^ and *NCoR1*^*ΔIECi*^ mice (*n* = 7–8 per group). (C) Growth curves of *NCoR1*^*f/f*^ mice and *NCoR1*^*ΔIECi*^ mice during HFD feeding (*n* = 8). (D) OGGT (2 g/kg BW) before tamoxifen induction and AUC analysis (*n* = 8). (E) OGGT (2 g/kg BW) after tamoxifen induction and AUC analysis (*n* = 8). (F) ITT (0.4 U/kg BW) after tamoxifen induction and AUC analysis (*n* = 8). (G) Plasma TG and TC levels in fasting state (*n* = 6). (H) Liver weight-to-body weight ratio (*n* = 7–8). (I) Fasting plasma HDL-c and LDL-c levels (*n* = 6). (J) RT‒qPCR analysis of *Ppara* and its target genes in the ileum. (*n* = 7–8). (K) Morphology of intestinal epithelium as shown by H&E staining and quantification of the villus length in the ileum (*n* = 5). (L) RT‒qPCR analysis of FXR and LXR target genes in the ileum (*n* = 7–8). (M) Total levels of plasma BAs, levels and ratio of 12*α*-hydroxylated and non-12*α*-hydroxylated BAs (*n* = 7). (N) RT-qPCR analysis of hepatic bile acid synthases and *Scd1* in HFD-fed *NCoR1*^*f/f*^ and *NCoR1*^*ΔIECi*^ mice (*n* = 6–7). Experimental data are expressed as the mean ± SEM. Two-tailed unpaired Student's *t* test was used for statistical analysis. Statistical significance was expressed as ∗*P* < 0.05, ∗∗*P* < 0.01, ∗∗∗*P* < 0.001.

To assess the therapeutic effect of NCoR1 deletion in IECs on obesity and metabolic syndrome after disease onset, we generated inducible NCoR1-knockout mice (*NCoR1*^*ΔIECi*^ mice) utilizing vil-Cre-ER^T2^ mice^36^. After 6 weeks of HFD feeding, tamoxifen was injected intraperitoneally for 5 days to induce NCoR1 deletion (Fig. 7A and B). Before injection, the *NCoR1*^*f/f*^ and *NCoR1*^*ΔIECi*^ mice gained similar weight during HFD feeding, and their oral glucose tolerance was also similar (Fig. 7C and D). After injection, the induced deletion of NCoR1 in IECs prevented weight gain in *NCoR1*^*ΔIECi*^ mice and improved insulin resistance and glucose tolerance compared to *NCoR1*^*f/f*^ mice (Fig. 7C, E and F). The effects of the induced deletion were still rather evident 7 weeks later, despite the gradual loss of the action of tamoxifen due to IEC self-renewal. The levels of plasma lipids, such as TG, TC and LDL-c, were also reduced, and the liver weight was significantly lower (Fig. 7G–I). Similar to findings observed in NCoR1 IKO mice, tamoxifen-induced NCoR1 deletion upregulated *Ppara* and its target genes in pathways such as ketogenesis (*Hmgcs2*), pyruvate metabolism (*Pdk4*), FA oxidation (*Acaa1b* and *Acsl1*), and lipid droplet formation (*Plin2*), and the villus length of the ileum was also increased in the *NCoR1*^*ΔIECi*^ mice (Fig. 7J and K). Moreover, induced NCoR1 deletion derepressed LXR signaling, reduced hydrophobic 12*α*-OH BAs levels, and FXR signaling remained unaffected (Fig. 7L–N). Collectively, these results showed that the beneficial effects of NCoR1 IKO can be achieved after disease onset.

## 4 Discussion

Our study revealed that intestinal-specific deletion of the corepressor NCoR1 induced robust improvements in obesity and metabolic syndrome by simultaneously decreasing the energy/lipid intake and enhancing the energy expenditure. The impairment of duodenum absorption mimicked the effects of metabolic surgery, while the stimulation of thermogenesis by succinate production in IECs provided a new link between the gut and BAT.

Notably, the levels of circulating succinate in the IKO mice showed dynamic changes during the fed and fasted states. Only the postprandial production of succinate was increased (Fig. 3C), whereas the fasting succinate levels were significantly reduced (Fig. 3E). Elevated fasting plasma succinate levels were previously reported to be associated with metabolic disorders, including T2D, obesity, and cardiovascular disease, and have been proposed to serve as a biomarker for poor metabolic status^37^^,^^38^. However, the circulating succinate levels are also increased during exercise and decreased at rest^39^^,^^40^. Metabolic surgery in obese patients not only reduces the fasting plasma succinate levels but also increases the meal/oral glucose-stimulated plasma succinate levels^41^. The plasma succinate levels were determined by both its production and elimination. Normally, succinate produced by the intestine after nutrient ingestion and by muscle during exercise gives rise to its circulating levels, whereas BAT selectively sequesters succinate from the circulation and decreases its levels. However, in the obese state, the production of succinate is enhanced by excess fat/nutrient intake, and its elimination is reduced due to BAT mass loss and dysfunction^42^; thus, its plasma levels were increased in obese conditions. As such, these studies suggested that the dynamic change in the succinate levels during fasting and refeeding, rather than the fasting levels, may predict a healthy state.

Although succinate supplementation improved metabolic syndrome, the thermogenic effects of succinate may only make a difference when its concentration is above a certain threshold. In the study conducted by Mills et al.^18^, 1% succinate administration from the start of HFD feeding didn't the body weight, but 1.5% and 2% succinate had robust effects. In another study^43^, 0.75 mg/mL (0.075%) succinate also had almost no effect on the metabolic status. In our study, we began to administer succinate to obese mice at the 24th week of high-fat feeding, and 1.5% succinate failed to induce any changes in the body weight, whereas 2.5% succinate significantly decreased the body weight of the WT mice. Thus, we speculated that although the succinate levels in obese patients or animals were high, they were still below the threshold needed to restore the functionality of impaired BAT. Like in patients received metabolic surgery, the postprandial plasma succinate levels were significantly higher than both the postprandial and fasting succinate levels in obese state^41^.

In this study, Intestinal NCoR1 deficiency activated PPAR*α* signaling in the intestine, but not in the liver and adipose tissue. The anti-obesity effects of intestinal PPAR*α* activation were in line with the effects of PPAR*α* agonists administration^6^. However, intestinal PPAR*α* deletion was also found to be reducing FA uptake and improving liver steatosis^5^^,^^23^^,^^24^. A closer look at the changes in PPAR*α* pathways revealed that intestinal PPAR*α* deficiency only significantly reduced the expression of FA transporters, but not of the genes in FA oxidation, ketogenesis and the tricarboxylic cycles (Fig. S5A and S5B), which may be explained by some overlapping functions between three PPAR isoforms. Thus, the production of succinate may still be similar between WT and intestinal PPAR*α* knock out mice, but the inhibition of FA uptake reduced the absorption of dietary fat. Whereas in the case of intestinal NCoR1 deletion, FA transporters were upregulated in the ileum, but the additional de-repression of LXR led to increased cholesterol excretion, which changed the composition of BAs (Fig. 6A–F) and impaired lipid absorption in the duodenum (Fig. 6I). The beneficial effects of disrupting duodenal lipid absorption have been observed with both metabolic surgery and transient duodenum depletion treatments^44^. Thus, the pro-absorption effects of PPAR*α* were avoided in intestinal NCoR1 deficient mice.

In our study, we only observed the beneficial effect of intestinal NCoR1 deficiency in the HFD-fed mice, but not in chow-fed mice. Probably because chow-fed mice are already lean and metabolically healthy compared to HFD-fed mice. Another reason would be that during HFD feeding, ligands (fatty acids and cholesterol derives) for nuclear receptors (NRs) are abundant, and without the “handbraking” function of NCoR1, the NRs are hyperactivated (PPAR*α*) or de-repressed (LXR).

Due to the important role of the gut in energy homeostasis, the approved treatments for obesity generally target intestine- or intestine-derived molecules. At present, the most effective treatment is metabolic surgery^45^, in which a large portion of the stomach and/or proximal small intestine is removed or bypassed to reduce the intestinal absorption of nutrients and appetite^46^^,^^47^. Obesity-related metabolic disorders, including T2D, dyslipidemia, and hepatic steatosis, are all greatly improved. Some patients do not even need drugs after metabolic surgery. However, the complexity of this surgical procedure has limited its wide use. Another promising class of drugs includes incretins and their analogs, such as semaglutide^48^ and tirzepatide^49^. They inhibit appetite, show great efficacy in reducing weight but are also linked with gastrointestinal adverse effects and thyroid tumors. The last drug class is lipase inhibitors, such as orlistat, which impaired the digestion of dietary fat in the gastrointestinal tract^25^, but adverse side effects such as oily spotting/stool render them less desirable for certain patients. As obesity has become more common, new anti-obesity modulators are needed to fulfil the needs of various patients. Our study showed that intestinal NCoR1 deficiency didn't affect appetite but showed a robust effect on metabolic syndrome by regulating both energy expenditure and lipid intake. Therefore, intestinal NCoR1 may serve as a potential new drug target for treating obesity and metabolic syndrome.

## 5 Conclusions

Metabolic surgery and GLP-1R drugs provided great options for patients with obesity and metabolic syndrome. However, there are still limits in their usage and novel treatments need to be developed. The deletion of NCoR1 in IECs activated PPAR*α* and LXR, and together, these two signaling pathways changed the energy balance to reduce anabolism and increase catabolism, thereby alleviating obesity and metabolic syndrome. Collectively, our findings may provide new insights for future development of metabolic syndrome treatments.

## Author contributions

Shaocong Hou: Writing – review & editing, Writing – original draft, Visualization, Validation, Methodology, Investigation, Data curation, Conceptualization. Hengcai Yu: Writing – original draft, Visualization, Validation, Methodology, Investigation, Data curation. Caihong Liu: Validation, Investigation, Data curation. Andrew M.F. Johnson: Methodology, Investigation, Conceptualization. Xingfeng Liu: Visualization, Methodology. Qian Jiang: Methodology, Investigation. Qijin Zhao: Methodology, Data curation. Lijuan Kong: Methodology, Investigation. Yanjun Wan: Validation, Investigation. Xiaowei Xing: Investigation. Yibing Chen: Visualization. Jingwen Chen: Investigation. Qing Wu: Methodology, Investigation. Peng Zhang: Resources, Project administration. Changtao Jiang: Supervision, Resources. Bing Cui: Supervision, Funding acquisition. Pingping Li: Writing – review & editing, Supervision, Resources, Project administration, Methodology, Funding acquisition, Data curation, Conceptualization.

## Conflicts of interest

The authors declare no conflicts of interest.

## Appendix A Supporting information

The following is the Supporting Information to this article:
