# Supplementary material for: Intestinal epithelial cell NCoR deficiency ameliorates obesity and metabolic syndrome
Source: Acta Pharm Sin B. 2024 Oct 10;14(12):5267–85. doi: 10.1016/j.apsb.2024.09.019 (PMC11725135; doi:10.1016/j.apsb.2024.09.019)
Supplement: Multimedia component 1 [file mmc1.pdf]

Supporting Information for

Original article

Intestinal epithelial cell NCoR deficiency ameliorates obesity and metabolic syndrome

Shaocong Hou[a,b,c,†], Hengcai Yu[a,b,c,†], Caihong Liu[a,b,c,†], Andrew M.F. Johnson[f], Xingfeng Liu[a,b,c], Qian Jiang[a,b,c], Qijin Zhao[a,b,c], Lijuan Kong[a,b,c], Yanjun Wan[a,b,c], Xiaowei Xing[a,b,c], Yibing Chen[a,b,c], Jingwen Chen[a,b,c], Qing Wu[d], Peng Zhang[e,‡], Changtao Jiang[d,‡], Bing Cui[a,c,‡], Pingping Li[a,b,c,‡,*]

[a]*State Key Laboratory of Bioactive Substance and Function of Natural Medicines, Institute of Materia Medica, Chinese Academy of Medical Sciences and Peking Union Medical College, Beijing 100050, China*

[b]*Diabetes Research Center of Chinese Academy of Medical Sciences, Beijing 100730, China*

[c]*CAMS Key Laboratory of Molecular Mechanism and Target Discovery of Metabolic Disorder and Tumorigenesis, Beijing 100050, China*

[d]*Department of Physiology and Pathophysiology, School of Basic Medical Sciences, Peking University, Beijing 100191, China*

[e]*Division of Metabolic and Bariatric Surgery, Department of General Surgery, Beijing Friendship Hospital, Capital Medical University, National Clinical Research Center for Digestive Diseases, Beijing 100050, China*

[f]*The Access to Advanced Health Institute, Seattle, Washington 98102, USA*

Received 27 March 2024; received in revised form 15 July 2024; accepted 14 September 2024

[*]Corresponding authors.

E-mail addresses: zhangpg@yahoo.com (Peng Zhang), jiangchangtao@bjmu.edu.cn (Changtao Jiang), cuibing@imm.ac.cn (Bing Cui), lipp@imm.ac.cn (Pingping Li).

[†]These authors made equal contributions to this work.

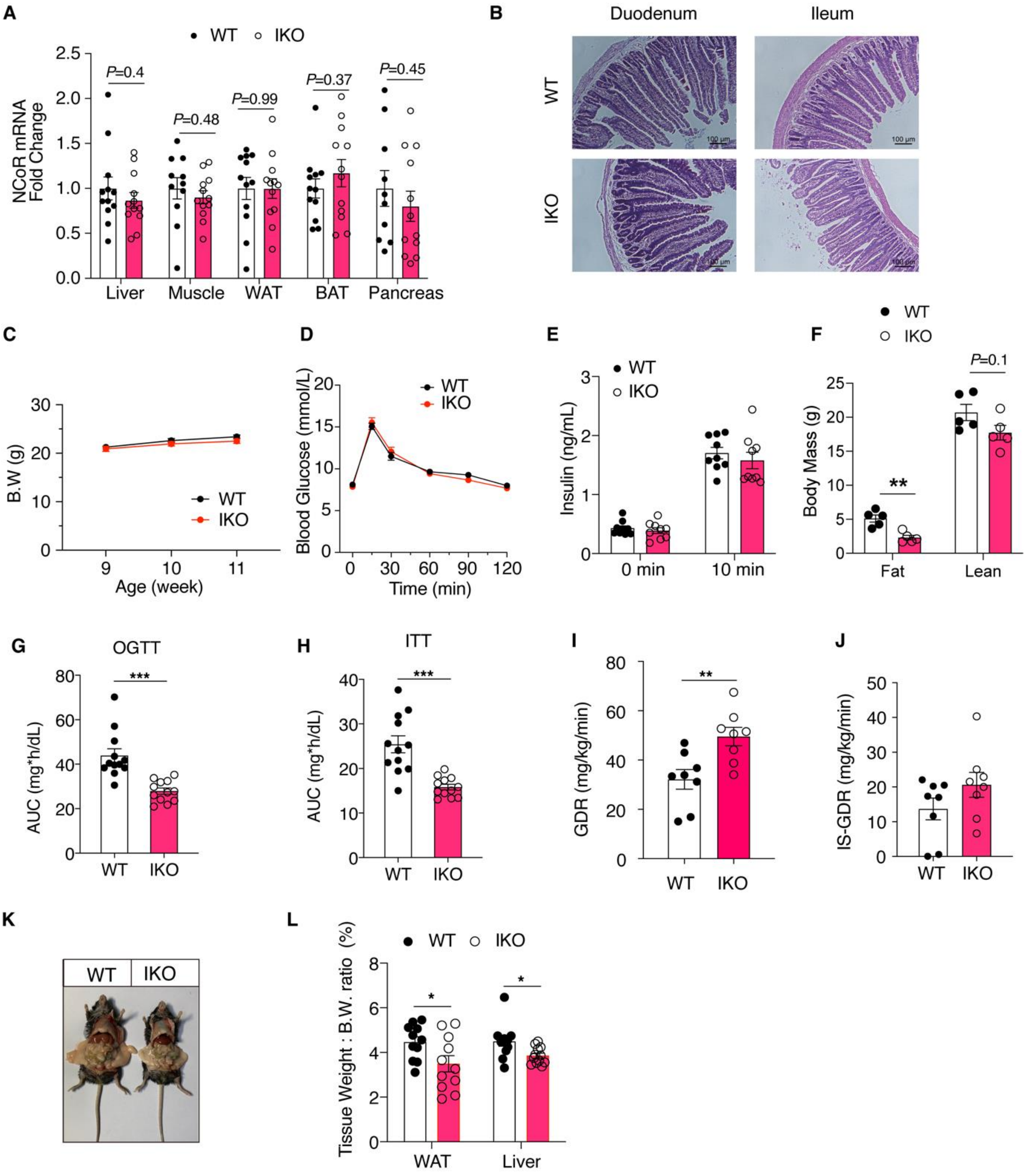

Figure S1 Conditional NCoR1 knockout in IECs did not affect chow diet-fed mice. (A) Relative expression of NCoR1 mRNA in other major metabolic tissues. *n* = 12. (B) H&E-stained duodenal and ileal tissues from HFD-fed WT and IKO mice. (C) Growth curves of WT and IKO mice fed with chow diet prior to HFD feeding. *n* = 12. (D) OGTT (2 g/kg BW) of WT and IKO mice fed with chow diet. *n* = 12. (E) Basal (0 min) and oral glucose-stimulated (10 min) plasma insulin levels in WT and IKO mice fed with chow diet. *n* = 9. (F) Fat mass and lean mass of HFD fed WT and IKO mice. *n* = 5. (G) Area under the curve (AUC) of OGTT, related to Fig. 1G. *n* = 12. (H) AUC of ITT, related to Fig. 1H. *n* = 12. (I, J) Related to Figure 1 (J–L). (I) Glucose disposal rate. (J) The insulin-stimulated glucose disposal rate (IS-GDR), which represents the insulin sensitivity of skeletal muscle. *n* = 9. (K) Representative images of

the livers and adipose tissue. (L) WAT, liver, and pancreas tissue weight-to-body weight ratios, related to Fig. 1P. n = 11–12. Experimental data are expressed as the mean ± SEM. Two-tailed unpaired Student's t test was used for statistical analysis. Statistical significance was expressed as $*P < 0.05$, $**P < 0.01$, $***P < 0.001$.

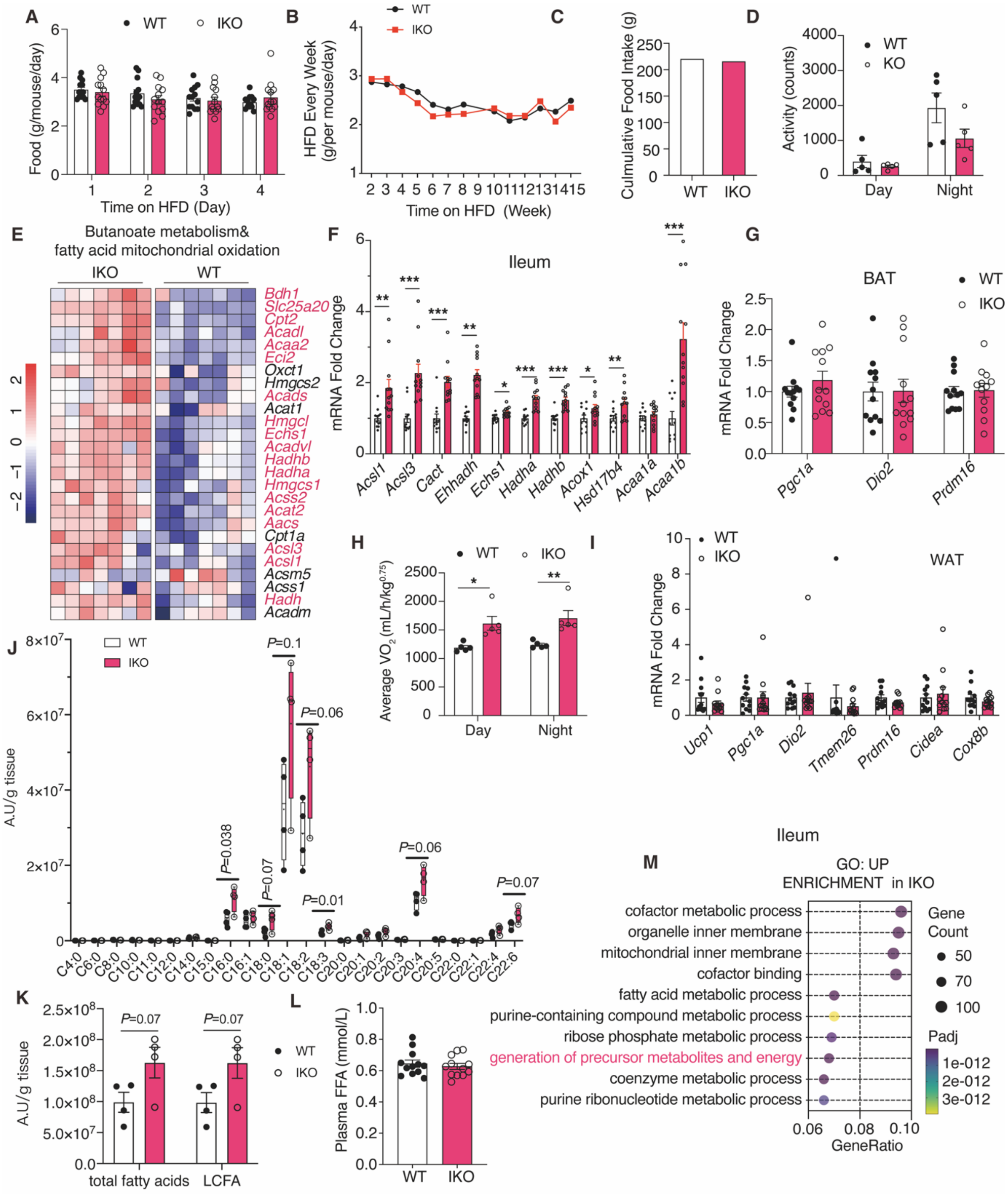

Figure S2 Intestinal NCoR1 deletion did not affect food intake or the expression of thermogenic genes in adipose tissue or muscle, but upregulated PPARα signaling in the ileum.

(A) Food intake of WT and IKO mice in the first 4 days after the diet was switched from a chow diet to an HFD. n = 13 per group. (B) Average daily food intake of WT and IKO mice during HFD feeding. n = 10–12. (C) Cumulative food intake. n = 10–12. (D) Locomotor activity of HFD-fed WT and IKO mice measured by a Columbus CLAMS. n = 5. (E) Heatmap showing genes involved in ketogenesis (butanoate) and the FA beta-oxidation pathway in the ileum of HFD-fed WT and IKO mice. Significantly upregulated genes are marked in red. n = 7. (F) RT-qPCR analysis of PPARα target genes controlling mitochondrial and peroxisomal oxidation of FAs in the ileum. n = 12. (G) mRNA expression of thermogenic genes in the BAT. n = 12. (H) Average oxygen consumption rate, related to Fig. 2B. n = 5. (I) mRNA expression of thermogenic genes in the WAT. n = 12. (J) Relative abundance of FFAs in the BAT of fasted WT and IKO mice. n = 4. (K) Relative abundance of total and long-chain FAs in the BAT of fasted WT and IKO mice. n = 4. (L) Fasting plasma FFA levels. n = 12. (M) Gene Ontology (GO) enrichment analysis of upregulated genes. The top 10 pathways enriched in IKO mice are shown. n = 7. Experimental data are expressed as the mean ± SEM. Two-tailed unpaired Student's t test or the R package DESeq2 was used for statistical analysis. Statistical significance was expressed as $*P < 0.05$, $**P < 0.01$, $***P < 0.001$.

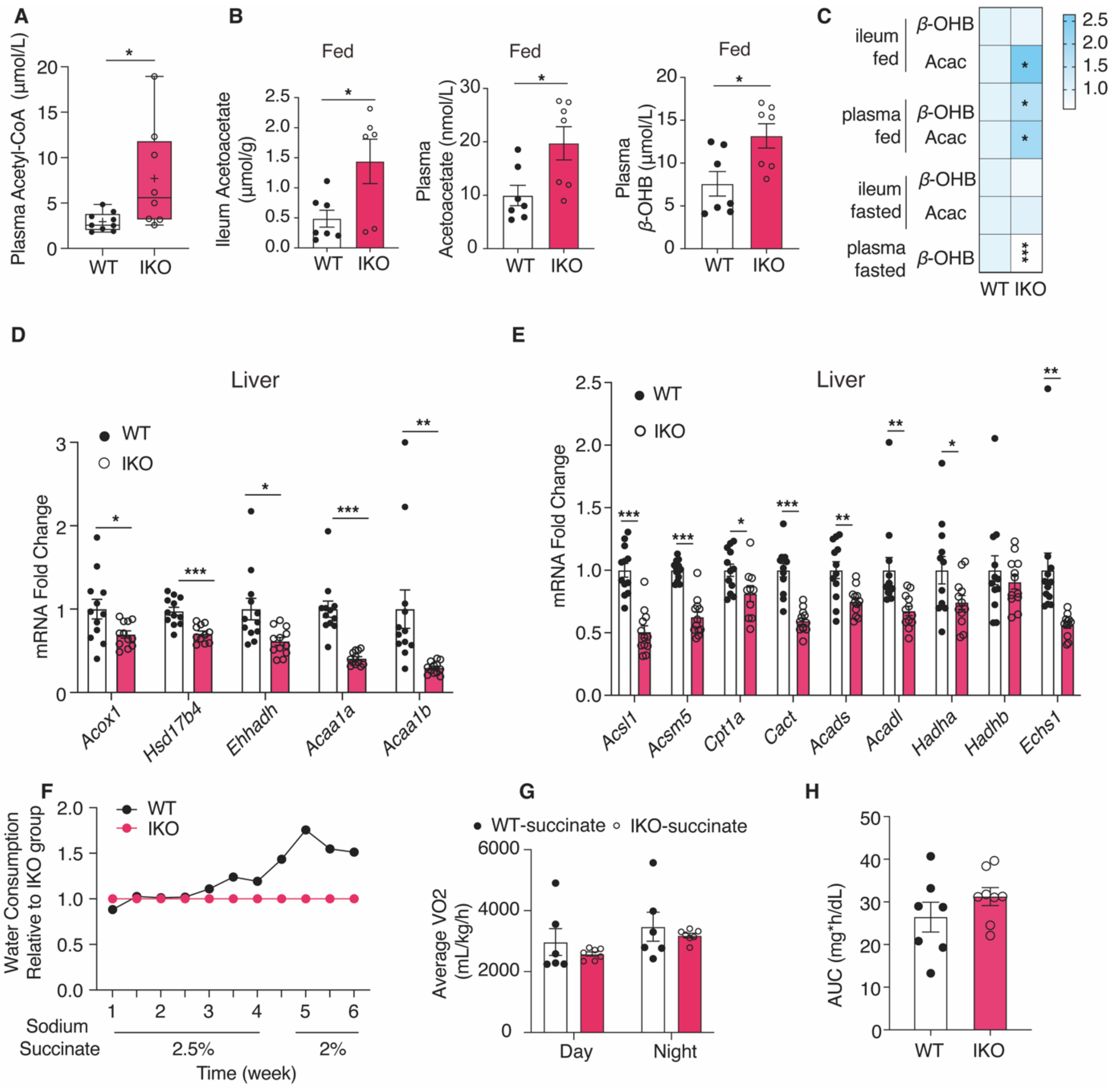

Figure S3 Intestinal NCoR1 deficiency increased postprandial succinate production and inhibited hepatic PPARα signaling. (A)Plasma Acetyl-CoA levels of HFD-fed WT and IKO mice in the fasting state. n = 8. (B) Ileal and plasma acetoacetate levels and plasma beta-hydroxybutyrate levels after 6 h of refeeding (Figure 3G). n =6–7. (C) Heatmap showing the ketone body abundances in the ileum and plasma of HFD-fed WT and IKO mice in the fasted and refed states. The abundances were expressed as percentages relative to levels in WT mice. n = 6–7. (D) Hepatic mRNA expression of PPARα target genes in peroxisomal fatty acid beta-oxidation pathways in HFD-fed WT and IKO mice. n = 12. (E) Hepatic mRNA expression of PPARα target genes in mitochondrial fatty acid beta-oxidation pathways. n = 12. (F) Consumption of drinking water by HFD-fed WT and IKO mice during sodium succinate administration. (G) Average oxygen consumption rate, related to Fig. 3I. n = 6. (H) AUC of OGTT, related to Fig. 3K. n = 7–8. Experimental data are expressed as the mean ± SEM. Two-tailed unpaired Student's t test was used for statistical analysis. Statistical significance is expressed as $*P < 0.05$, $**P < 0.01$, $***P < 0.001$.

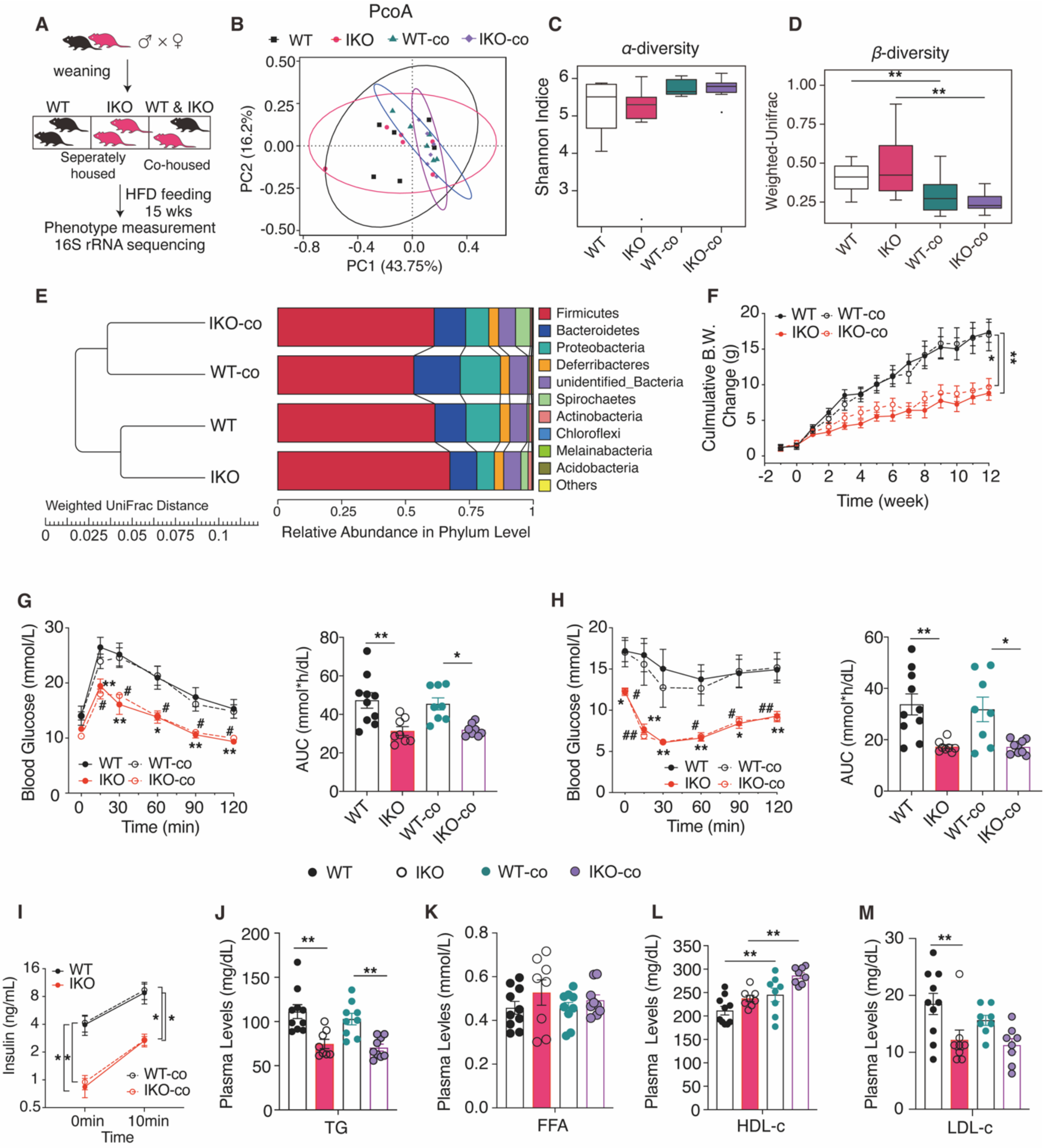

Figure S4 The effects of intestinal NCoR1 deficiency were not mediated by the gut microbiota. (A) Experimental design of the cohousing experiment. WT, IKO, WT-co and IKO-co mice were fed with HFD. Feces from the 13th week were sequenced for 16S rRNA in (B–E). OGTTs (G) were performed after 10 weeks of feeding, ITTs (H) were performed after 12 weeks of feeding, and the plasma lipid profiles (J–M) were analyzed after the mice were sacrificed. (B) Principal coordinate analysis of 16S rRNA sequencing data. $n = 6$. (C) Alpha diversity between separately housed and cohoused HFD-fed WT and IKO mice, as indicated by the Shannon indices. $n = 6$. (D) The beta diversity of the gut microbiota in HFD-fed WT, IKO, WT-co and IKO-co mice was analyzed by a Wilcoxon signed-rank test of weighted UniFrac distances. $n = 6$. (E) Weighted UniFrac distances and relative abundances of the HFD-fed WT, IKO, WT-co and IKO-co mice at the phylum level. $n = 6$. (F) Changes in the body weights of separately

housed (WT, IKO) and cohoused mice (WT-co, cohoused WT mice; IKO-co, cohoused IKO mice) after HFD feeding. n = 8–10. (G) OGTT (2 g/kg BW) and AUC analysis. n = 8–10. (H) ITT (0.3 U/kg BW) and AUC analysis. n = 8–10. (I) Plasma insulin levels at fasting state and 10 min post-oral glucose loading. n = 8–10. (J) Fasting plasma TG levels. n = 8–10. (K) Fasting plasma FFA levels. n = 8–10. (J) Fasting plasma HDL-c levels. n = 8–10. (K) Fasting plasma LDL-c levels. n = 8–10. Experimental data are expressed as the mean ± SEM. Wilcoxon signed-rank test (B–E) and two-way ANOVA with Tukey's correction for multiple comparisons (F–M) were used for statistical analysis. Statistical significance was expressed as $^{*}P < 0.05$ and $^{**}P < 0.01$ between the indicated groups. In (G, H), $^{*}P < 0.05$ and $^{**}P < 0.01$ for WT *vs*. IKO, and $^{\#}P < 0.05$ and $^{\#\#}P < 0.01$ for WT-co *vs*. IKO-co.

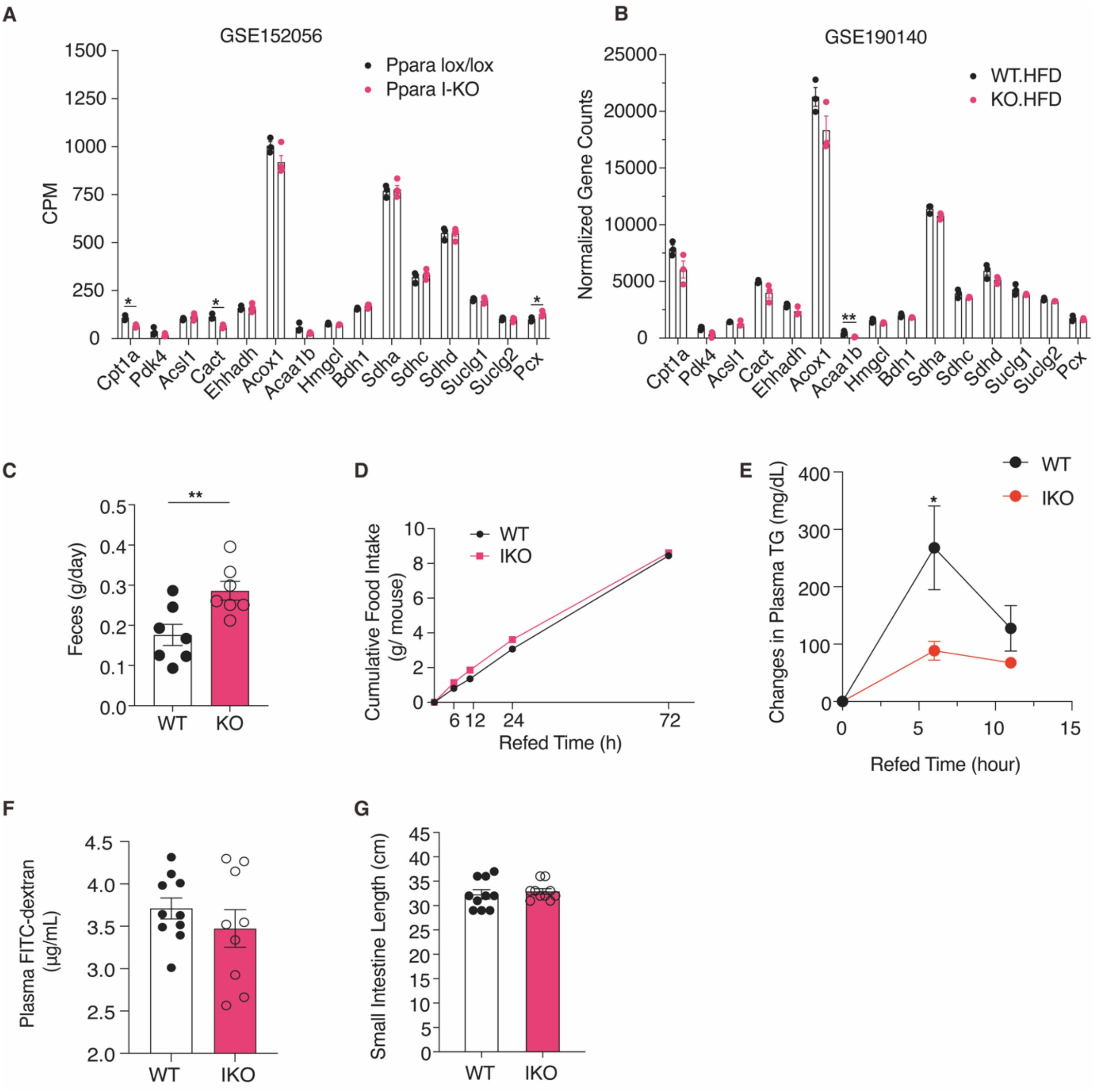

Figure S5 NCoR1 deficiency in IECs impaired lipid absorption but did not change the intestinal permeability or small intestine length. (A) Duodenal expression of genes participated

in FA oxidation and the tricarboxylic cycle in WT and intestinal PPARα knock-out mice fed with HFD for 8 weeks. Data from the GEO data series GSE152056. Normalized counts (CPM) were analyzed by edgeR library in R. n = 3. (B) Expression of genes participated in FA oxidation and tricarboxylic cycle in WT and intestinal PPARα knock-out mice fed with HFD for 12 weeks. Data from the GEO data series GSE190140. Normalizedgene counts were analyzed by DESeq2 package in R. n = 3–4. (C) Fecal output of HFD-fed WT and IKO mice over a 24-h period without correction for body weight. n = 7. (D) Cumulative food intake of WT and IKO mice during refeeding after overnight fasting. Related to Fig. 4I. (E) Changes in the basal plasma TG levels in HFD-fed (14-week-old) WT and IKO mice during the refed state after overnight fasting (15 h). n = 8. (F) Plasma FITC-dextran concentration measured by fluorescence 4 h after oral gavage. n = 9–10. (G) Small intestine length of HFD-fed WT and IKO mice. n = 10. Experimental data are expressed as the mean ± SEM or the means only. Unpaired two-tailed Student's t test (A-E), Welch's t-test (F) and DESeq2 (G) were used for statistical analysis. Statistical significance was expressed as *P < 0.05.

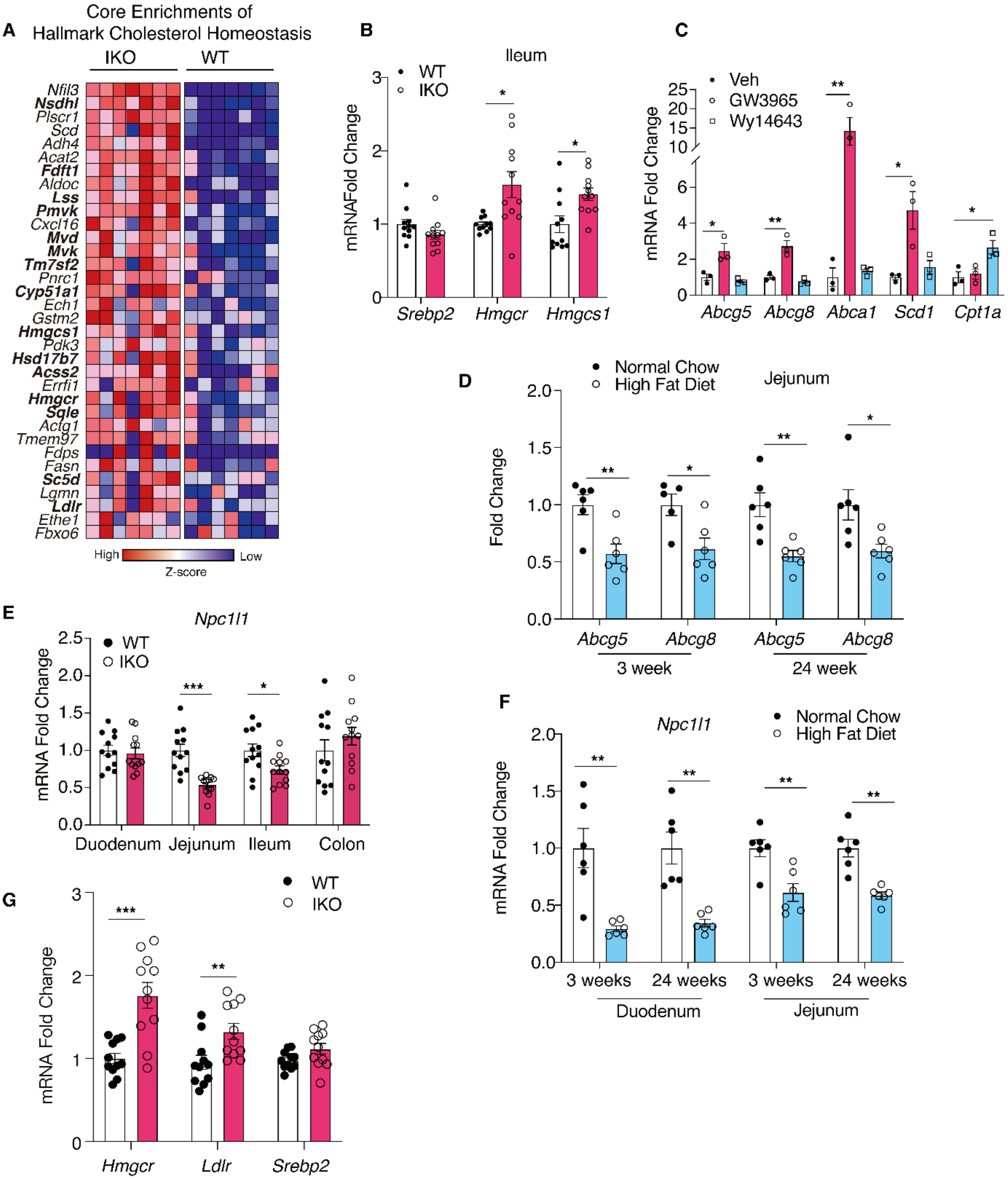

Figure S6 The cholesterol synthesis pathway was upregulated in the ileum and liver, and intestinal *Npc1l1* expression was reduced. (A) Heatmap showing the top-ranked genes identified by GSEA using the Hallmark cholesterol homeostasis gene set (related to Fig. 5B). Genes related to cholesterol biosynthesis (mevalonate pathway) are marked in bold. (B) mRNA expression of *Srebp2* and its target genes *Hmgcr* and *Hmgcs1* in the ileum of WT and IKO mice fed with HFD. $n = 11$. (C) Effects of LXR and PPARα agonists on the expression of their respective target genes. WT mouse small intestine organoids were treated with GW3965 (1 µmol/L) and Wy14643 (5 µmol/L) for 6 h (with three biological replicates for each treatment). (D) Expression of *Abcg5* and *Abcg8* mRNA in the jejunum of C57BL/6J mice at 3 weeks and 24 weeks after HFD/chow diet feeding. $n = 6$ per group. (E) RT-qPCR analysis of *Npc1l1* in

the intestines of WT and IKO mice fed with HFD for 15 weeks. $n = 12$. (F) mRNA expression of *Npc1l1* in the duodenum and jejunum of C57BL/6J mice at different time points after HFD/chow diet feeding. $n = 6$. (G) Hepatic mRNA expression of *Srebp2* and *Hmgr* in WT and IKO mice fed with HFD. $n = 11$. Experimental data are expressed as the mean ± SEM. The R package DESeq2 (A), one-way ANOVA with Dunnett's correction for multiple comparisons (B), and unpaired two-tailed Student's *t* test (C–G) were used for statistical analysis. Statistical significance is expressed as $*P < 0.05$, $**P < 0.01$, $***P < 0.001$.

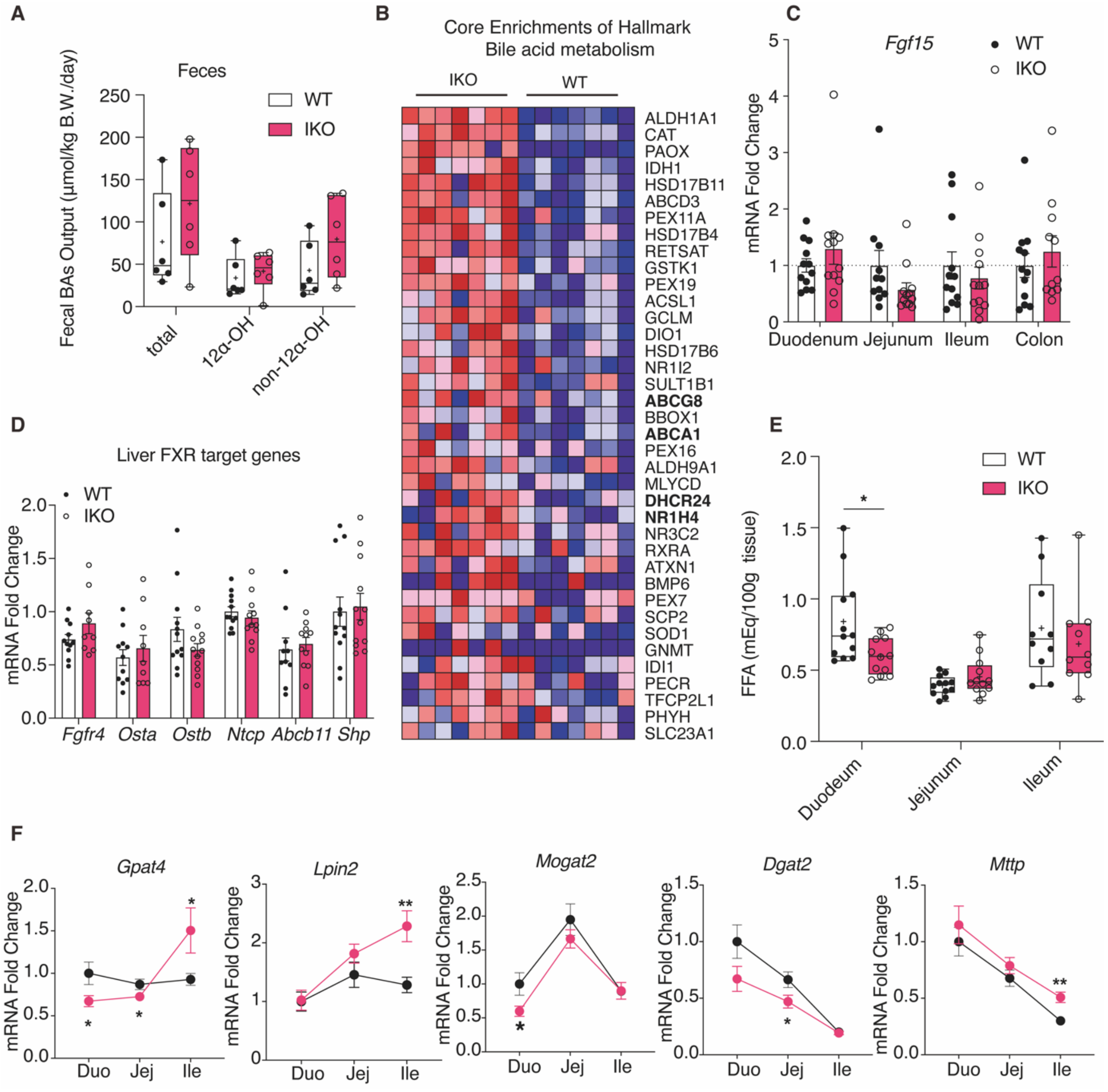

Figure S7 The effects of intestinal NCoR1 deletion on BA metabolism did not involve FXR signaling. (A) 24-h fecal output of total BAs, 12*α*-hydroxylated BAs, and non-12*α*-hydroxylated BAs (corrected for the body weight) in WT and IKO mice fed with HFD for 15 weeks. $n = 6$. (B) Heatmap showing the core enrichment genes identified by GSEA using the hallmark BA metabolism gene set (related to Fig. 5B). $n = 7$. (C) RT-qPCR analysis of *Fgf15*

in the small intestine and colon of WT and IKO mice fed with HFD for 15 weeks. n = 11–12. (D) Expression of *Fgfr4* and FXR target genes in the liver of WT and IKO mice fed with HFD for 15 weeks. n = 11–12. (E) FFA concentrations in the duodenum, jejunum, and ileum of WT and IKO mice fed with HFD for 15 weeks. n = 12 for the duodenum and jejunum, n = 10 for the ileum. (F) Expression of genes responsible for TG synthesis and export in the small intestine. n = 11–12. Experimental data are expressed as the mean ± SEM. GSEA and an unpaired two-tailed Student's t test were used for statistical analysis. Statistical significance is expressed as *$P < 0.05$, **$P < 0.01$, ***$P < 0.001$.

Table S1 Primers.

**Mouse genotyping primers**	**Sequence (5′ to 3′)**	**Sequence (5′ to 3′)**
NCoR	TTGGCCTTGGAGTAAATGCTGTGAG	GGAAACTACCTACCTGAATCCATGG
Villincre	GCATTACCGGTCGTAGCAACG	GAACGCTAGAGCCTGTTTTGC
**Mouse qPCR primers**	**Sequence (5′ to 3′)**	**Sequence (5′ to 3′)**
36b4	AGATTCGGGATATGCTGTTGGC	TCGGGTCCTAGACCAGTGTTC
Abca1	AAAACCGCAGACATCCTTCAG	CATACCGAAACTCGTTCACCC
Abcb11	TCTGACTCAGTGATTCTTCGCA	GTGTAGAGTGAAGTCCTCCTTAGC
Abcg5	AGGGCCTCACATCAACAGAG	GCTGACGCTGTAGGACACAT
Abcg8	CTGTGGAATGGGACTGTACTTC	GTTGGACTGACCACTGTAGGT
Acaa1a	TCTCCAGGACGTGAGGCTAAA	CGCTCAGAAATTGGGCGATG
Acaa1b	CAGGACGTGAAGCTAAAGCCT	CTCCGAAGTTATCCCCATAGGAA
Acads	CATCTCTTCCCCACAGCTCA	CAGGTAATCCAAGCCTGCAC
Acadl	TCTTTTCCTCGGAGCATGACA	GACCTCTCTACTCACTTCTCCAG
Acc	GGAGATGTACGCTGACCGAGAA	ACCCGACGCATGGTTTTCA
Acly	CTCACACGGAAGCTCCATAA	ACGCCCTCATAGACACCATC
Acox1	TGAGGCGCCAGTCTGAAATC	CCGTCTGCAGCATCATAACA
Acsl1	ACCAGCCCTATGAGTGGATTT	CAAGGCTTGAACCCCTTCTG
Acsm5	CCGATCCCTGAGGTGGTAG	GGTGCCCTGTCTTTTCCAG
Agpat2	CAGCCAGGTTCTACGCCAAG	TGATGCTCATGTTATCCACGGT
Asbt	GTCTGTCCCCCAAATGCAACT	CACCCCATAGAAAACATCACCA
Cact	GACGAGCCGAAACCCATCAG	AGTCGGACCTTGACCGTGT
Cd36	AGATGACGTGGCAAAGAACAG	CCTTGGCTAGATAACGAACTCTG
Cidea	TGACATTCATGGGATTGCAGAC	GGCCAGTTGTGATGACTAAGAC
Cox8b	TGTGGGGATCTCAGCCATAGT	AGTGGGCTAAGACCCATCCTG
Cpt1a	CTCCGCCTGAGCCATGAAG	CACCAGTGATGATGCCATTCT
Cyp27a1	CCAGGCACAGGAGAGTACG	GGGCAAGTGCAGCACATAG
Cyp2c70	TGGCTTTCTCAGCAGGAAGAA	AACTGGCTTGGTGTCGATGT
Cyp7a1	GGGATTGCTGTGGTAGTGAGC	GGTATGGAATCAACCCGTTGTC
Cyp7b1	GGAGCCACGACCCTAGATG	TGCCAAGATAAGGAAGCCAAC
Cyp8b1	CCTCTGGACAAGGGTTTTGTG	GCACCGTGAAGACATCCCC
Dgat2	GCGCTACTTCCGAGACTACTT	GGGCCTTATGCCAGGAAACT

Dio2	AATTATGCCTCGGAGAAGACCG	GGCAGTTGCCTAGTGAAAGGT
Echs1	AGCCTGTAGCTCACTGTTGTC	ATGTACTGAAAGTTAGCACCCG
Ehhadh	ATGGCTGAGTATCTGAGGCTG	GGTCCAAACTAGCTTTCTGGAG
Elovl3	TTCTCACGCGGGTTAAAAATGG	GAGCAACAGATAGACGACCAC
Elovl6	GAAAAGCAGTTCAACGAGAACG	AGATGCCGACCACCAAAGATA
Fabp1	GGGAAGAAAATCAAACTCACCATC	AGTTGTCACCATTTTATTGTCACC
Fabp2	TTGCTGTCCGAGAGGTTTCT	GCTTTGACAAGGCTGGAGAC
Fasn	GGAGGTGGTGATAGCCGGTAT	TGGGTAATCCATAGAGCCCAG
Fatp4	ACTGTTCTCCAAGCTAGTGCT	GATGAAGACCCGGATGAAACG
Fgf15	ATGGCGAGAAAGTGGAACGG	CTGACACAGACTGGGATTGCT
Fgfr4	GCTCGGAGGTAGAGGTCTTGT	CCACGCTGACTGGTAGGAA
Gpat4	AGCTTGATTGTCAACCTCCTG	CCGTTGGTGTAGGGCTTGT
Hadha	TGCATTTGCCGCAGCTTTAC	GTTGGCCCAGATTTCGTTCA
Hadhb	ACTACATCAAAATGGGCTCTCAG	AGCAGAAATGGAATGCGGACC
Hmgcr	AGCTTGCCCGAATTGTATGTG	TCTGTTGTGAACCATGTGACTTC
Hmgcs2	CCTGCAAGTGAAGAGAGCGA	TAGAAAACCTCTGCTGGGCTG
Hsd17b4	TGCAATACTCTCGCCATTGA	GCTTCAGGGCTTCAACAAGA
Ibabp(Fabp6)	CTTCCAGGAGACGTGATTGAAA	AACTTGTTGCTCATAATGTTGCC
Ldlr	AGGCTGTGGGCTCCATAGG	TGCGGTCCAGGGTCATCT
Lpin2	GAAGTGGCGGCTCTCTATTTC	AGAGGGTTACATCAGGCAAGT
Mogat2	TGGGAGCGCAGGTTACAGA	CAGGTGGCATACAGGACAGA
Mttp	CTCTTGGCAGTGCTTTTTCTCT	GAGCTTGTATAGCCGCTCATT
NCoR	TGCGTCAGCTTTCTGTGATTCCACC	TGATTTCTGCCTCTGCGTTTTCCAT
Npc1l1	GCAAGGTGATCAGGAGGTTGA	ATCCTCATCCTGGGCTTTGC
Ntcp	CAAACCTCAGAAGGACCAAACA	GTAGGAGGATTATTCCCGTTGTG
Osta	CCCTGACGGCATCTATGACC	TGGCTTGACGGAAAAGGATGG
Ostb	AGATGCGGCTCCTTGGAATTA	TGGCTGCTTCTTTCGATTTCTG
Pdk4	AGGGAGGTCGAGCTGTTCTC	GGAGTGTTCACTAAGCGGTCA
Pgc1a	TATGGAGTGACATAGAGTGTGCT	CCACTTCAATCCACCCAGAAAG
Plin2	GACCTTGTGTCCTCCGCTTAT	CAACCGCAATTTGTGGCTC
Pnliprp2	ATGCCTATGGATGTCCGTGGA	ATGCCTATGGATGTCCGTGGA
Prdml6	CAGCACGGTGAAGCCATTC	GCGTGCATCCGCTTGTG
Shp	TCTGCAGGTCGTCCGACTATTC	AGGCAGTGGCTGTGAGATGC
Scd1	TTCTTGCGATACACTCTGGTGC	CGGGATTGAATGTTCTTGTCGT
Srebf2	GGATCCTCCCAAAGAAGGAG	TTCCTCAGAACGCCAGACTT
Tmem26	TTCCTGTTGCATTCCCTGGTC	GCCGGAGAAAGCCATTTGT
Ucp1	AGGCTTCCAGTACCATTAGGT	CTGAGTGAGGCAAAGCTGATTT

Table S2 Key resources table.

REAGENT or RESOURCE	SOURCE	IDENTIFIER
Antibodies		
ABCG5	Proteintech	Cat# 27722-1-AP
ABCG8	Abcam	Cat# ab223056
ACLY	CST	Cat# 4332
FASN	CST	Cat# 3180
SCD1	CST	Cat# 2794
CD36	Santa Cruz	Cat#sc-7309
FABP1	Santa Cruz	Cat#sc-271591
Beta Actin	Proteintech	Cat# 66009-1-Ig
HSP90	Proteintech	Cat# 60318-1-Ig
Goat Anti-Mouse IgG(H+L)-HRP conjugated	Bioeasytech	BE0102-100
Goat Anti-Rabbit IgG(H+L)-HRP conjugated	Bioeasytech	BE0101-100
Chemicals, peptides, and recombinant proteins		
Insulin	Eli Lily and Company	Humulin R
D-[3-^{3}H] glucose	PerkinElmer	NET331C
DMEM	Gibco	11995-065
Advanced DMEM	Gibco	12634010
Matrigel	BD/Corning	356231
GlutaMAX-I	Thermo	35050061
EGF	Invitrogen	PMG8043
N2	Thermo	17502048
B27	Thermo	17504044
Noggin	R&D	6057-NG-100
R-spondin1	R&D	4645-RS-025
N-Acetylcysteine	Sigma-Aldrich	A9165
Y-27362	Sigma-Aldrich	Y0503
GW3965	Med Chem Express	HY-10627A
Wy14643	Med Chem Express	HY-16995
Poloxamer 407	Sigma-Aldrich	16758
Disodium succinate	MedChemExpress	HY-W015410
DMSO	Sigma-Aldrich	D4540
Feta Bovine Serum	Gibco	16000-044
FITC-Dextran	Sigma-Aldrich	FD-4
GLutaAMX	Gibco	35050-061
PBS	Monad	CR10201M
Penicillin/Streptomycin	Gibco	15140-122
Phosphatase Inhibitor A	Beijing bimake	B15001-A
Phosphatase Inhibitor B	Beijing bimake	B15001-B
Protease Inhibitor	Beijing bimake	B14001
Humulin	Lily and company	J20170043
RIPA lysis buffer	Beijing bimake	P0013B
SDS	Beijing Solarbio	S8010
SDS-PAGE sample buffer	EASYBIO	BE6230
Sitagliptin Phosphate	Merck Sharp & Dohme	H20140153
Skim milk powder	Oxoid Ltd.	LP0031
Tamoxifen	Sigma-Aldrich	T5648
TBST	Monad	130351
Trypsin-EDTA	Gibco	25200-056
TEMED	Beijing Applygen	A1006
Trans DNA marker I	Beijing TransGen	BM401-01
Tris-base	Beijing Solarbio	T8060
Triton X-100	Sigma-Aldrich	SLBN8333V
Trizol Reagent	Life Technologies	15596018
Tween-20	Beijing Coolaber	CT11551
Universal Antibody Diluent	New Cell & Molecular	WB500D
Critical commercial assays		

Insulin ELISA kit	Alpco	80-INSMSU-E10
Triglyceride Assay kit	Zhongsheng Beikong	20162400911
Serum Triglyceride Determination Kit	Sigma-Aldrich	TR0100
Total Cholesterol assay kit	Zhongsheng Beikong	20162400910
NEFA kit	FUJIFILM Wako Pure Chemical Corp.	294-63601
HDL and LDL/VLDL cholesterol assay kit	Cell Biolabs	STA-391
3-Hydroxy-Butyrate kit	Sigma-Aldrich	MAK041
2x SYBR Green qPCR Master Mix	Shanghai Bimake	B21203
BCA protein assay kit	Beijing TIANGEN	PA115-02
High-Capacity cDNA Reverse Transcription Kit	Thermo Fisher Scientific	4368813
ROS measurement kit	Bestbio	BB-470538
Oil Red O kit	Servicebio	G1015
High-sig ECL western blotting substrate	Shanghai Tanon	180-5001
Deposited data		
16S rRNA sequencing of feces	This paper	PRJNA995891
RNA sequencing of intestinal epithelial cells from ileum	This paper	GSE237833
Oligonucleotides		
See Supplementary table 1	This paper	NA
Experimental models: Cell lines		
Intestinal organoids	This paper	Mahe, M.M. et al [60]
Experimental models: Organisms/strains		
C57 BL 6/J	Vital River Lab Animal Technology	NA
Software and algorithms		
Image-J	National Institutes of Health (NIH)	https://imagej.nih.gov/ij/
Prism	Graphpad	Version 6.0
CLAMS	Oxymax	Columbus Instruments
R	https://www.r-project.org	Version 4.1.0
Other		
Rodent Diet with 60 kcal% Fat	Research Diets	Cat# D12492
Glucometer	Roche	Accuchek
Catheter	Dow Corning	Silastic 508-001
